# A Field Test of a Web-Based Workplace Health Promotion Program to Improve Dietary Practices, Reduce Stress, and Increase Physical Activity: Randomized Controlled Trial

**DOI:** 10.2196/jmir.9.2.e17

**Published:** 2007-06-19

**Authors:** Royer F Cook, Douglas W Billings, Rebekah K Hersch, Anita S Back, April Hendrickson

**Affiliations:** ^1^ISA AssociatesAlexandriaVAUSA

**Keywords:** Web-based, interventions, stress, diet, activity

## Abstract

**Background:**

Most work sites engage in some form of health promotion programming designed to improve worker health and reduce health care costs. Although these programs have typically been delivered through combinations of seminars and print materials, workplace health promotion programs are increasingly being delivered through the Internet.

**Objective:**

The purpose of this research was to evaluate the effectiveness of a Web-based multimedia health promotion program for the workplace, designed to improve dietary practices, reduce stress, and increase physical activity.

**Methods:**

Using a randomized controlled trial design with pretest-posttest comparisons within each group, 419 employees of a human resources company were randomly assigned to the Web-based condition or to a condition that provided print materials on the same topics. All subjects were assessed at pretest and posttest through an online questionnaire containing multiple measures of health behavior and attitudes. The test period was 3 months. Questionnaire data were analyzed mainly by analysis of covariance and *t* tests.

**Results:**

Retention rates were good for both groups—85% for the Web-based group and 87% for the print group. Subjects using the Web-based program performed significantly better than the print group on Attitudes Toward a Healthful Diet (F_1,415_ = 7.104, *P =* .008) and Dietary Stage of Change (F_1,408_ = 6.487, *P* = .01), but there were no significant group differences on the five other dietary measures. Both groups also showed improvement from pretest to posttest on most dietary measures, as indicated by significant *t* tests. Within the Web-based group, dosage analyses showed significant effects of the number of times the subject accessed the program on measures of Dietary Self-Efficacy (F_2,203_ = 5.270, *P* = .003), Attitudes Toward a Healthful Diet (F_2,204_ = 2.585, *P* = .045), and Dietary Stage of Change (F_2,200_ = 4.627, *P* = .005). No significant differences were found between the two groups on measures of stress or physical activity, although *t* tests of pretest-posttest changes indicated that both groups improved on several of these measures. The Web-based group gave significantly higher ratings to the program materials than the print group on all health topics and in their overall evaluation (F_1,410_ = 9.808, *P* = .002).

**Conclusions:**

The Web-based program was more effective than print materials in producing improvements in the areas of diet and nutrition but was not more effective in reducing stress or increasing physical activity. The higher ratings given to the Web-based program suggest that workers preferred it to the print materials. Both groups showed numerous pretest-posttest improvements in all health topics, although such improvements might be attributable in part to a Hawthorne effect. Results suggest that a multimedia Web-based program can be a promising means of delivering health promotion material to the workforce, particularly in the area of diet and nutrition.

## Introduction

Workplace health promotion activities and modes of delivery vary widely, from ad hoc events, such as health fairs and provision of print materials, to comprehensive programs involving health risk appraisals, preventive interventions addressing fitness and dietary practices, and intensive disease management programs. A recent comprehensive review of the clinical efficacy and cost-effectiveness of work site comprehensive health promotion and disease management programs concluded that “studies to date indicate positive clinical and cost outcomes” [1]. Although this assessment specifically refers to *comprehensive* programs that focus on risk reduction (particularly for high-risk employees), the generally positive conclusion is congruent with other studies demonstrating effectiveness and cost savings from health promotion and disease management programs at work sites [2,3].

Most of the research conducted to date on work site health has focused on traditional approaches involving in-person (group or individual) interventions, often supplemented with video and print materials. However, computer-based interventions are beginning to emerge, in the workplace as well as in the home, spurred by the tremendous growth in access to the Internet and in the creation of health improvement programs available on the Web. In a review article, Evers points out that The Pew Internet & American Life Project now splits Internet access in the United States into three tiers: those who are truly offline (22% of adults), those with modest connections such as dial-up (40%), and those who are the highly wired broadband elite (33%) [4]. Workplaces are rapidly joining this “elite” group, and the Internet has become increasingly used as a channel for health interventions. Evers notes that despite the familiar caveats about the emerging nature of the data on the effectiveness of Web-based interventions, there is increasing excitement about the potential for Internet technology to facilitate the development of interactive, tailored, multimedia behavior change programs [4]. Of particular note are the results of a study by Wantland and associates, whose meta-analysis of 22 studies found that Web-based health interventions demonstrated improved outcomes over non-Web-based interventions [5]. And in a recent evaluation of a Web-based training program for health promotion practitioners, our research team found the program to be more effective than print materials [6]. Yet, despite the proliferation of Web-based health improvement programs in the workplace, in comparison to traditional modes of delivery there has been relatively little evaluation of Web-based workplace interventions, particularly preventive interventions targeting multiple health behaviors.

The purpose of this study was to test the efficacy of a Web-based health promotion program for the workplace designed to improve participants’ dietary practices, increase their level of physical activity, and reduce their stress. The research sought to (1) compare the program’s effectiveness with a print-based intervention; (2) assess the program’s effectiveness in achieving positive changes in multiple self-report measures of health status, attitudes, and practices; and (3) assess participants’ reactions to the Web-based program compared to the print materials.

It should be understood that the purpose of the research was *not* to compare material presented in the Web-based format with the exactly same material presented in print. Instead, the purpose was to test the efficacy of a multimedia Web-based program (created in Macromedia Flash) compared to high-quality commercially available print materials on the same topics (but not necessarily the same content) as a control.

This research represents a continuation of our group’s research on the development and testing of video-centered programs designed to improve workforce health and reduce substance abuse [7-11]. As with the video-centered programs, the present program was shaped by a social-cognitive conceptual model based mainly on the work of Bandura [12,13] and emphasizing observational learning, boosting of self-efficacy, and self-regulation. This model was first adapted to workforce health by the authors of a 1990 monograph [14] and was further refined as suggested by the data from subsequent studies (see [9] for the most recent discussion of the model). In addition to social-cognitive theory, the model also draws on the transtheoretical model of Prochaska and associates [15], recognizing that individuals are typically at one of several stages in their readiness to change health behaviors, that interventions must be appropriate to their stage of change, and that moving individuals from one stage to another can be as important as effecting behavior change.

The central hypotheses of the study were as follows:

1. The Web-based program group will show significantly greater improvement in the outcome measures of diet, stress, and physical activity than the print group.
2. The Web-based program group will show significant improvement in the outcome measures from pretest to posttest.
3. The Web-based program will be rated as more informative and appealing than the print materials.

## Methods

### Design

The study was a randomized controlled trial with pretest-posttest comparisons in each group. Employees in three offices of a human resources company were recruited and randomly assigned to the Web-based program condition or the print condition (receiving print materials covering the same health topics as the Web-based program). It was recognized that the print materials could also be effective instruments of health behavior improvement (unlike a no-treatment control group) and could be a challenge as a control group. Indeed, studies have found print materials to be effective in improving behaviors and outcomes related to walking and other physical activities [16,17], alcohol problems [18], and emotional disorders [19]. However, we believed that the distribution of print materials would be a likely workplace alternative to an online program; therefore, the print group was thought to be an appropriate control group for the study.

The field test was conducted at a major human resources provider with a workforce of approximately 5000 employees. The test was conducted at three of the company’s offices located in Atlanta, GA, Minneapolis, MN, and Fountain Valley, CA. The study took place from August 1, 2004 until July 31st, 2005, with recruitment starting in January 2005 and data collection ending in June 2005. Recruitment procedures included an email letter from management describing the purpose and nature of the field test and the program, as well as the incentives for participating (US $50/survey and a US $500 raffle prize). An online flyer was attached to the email, presenting the basic information in a more graphic, colorful fashion. In addition, posters describing the program and the field test were placed throughout the participating offices at the start of participant recruitment. The recruitment period lasted 3 weeks, followed by the pretest of all participants.

The pretest and posttest surveys were online survey assessments containing multiple self-reports of health practice and attitudes (described below). The pretest period lasted 2 weeks. Following this, the Web-based group was provided with codes to access the online program, and the print group was provided with a packet of print materials. The test period lasted 3 months, followed by the posttest survey of all participants.

The study methods and procedures were approved and periodically reviewed by the Institutional Review Board of ISA Associates, which has a Federal Wide Assurance from the Office of Human Research Protections of the National Institutes of Health.

### Sample

A total of 480 employees participated in the pretest assessment, and 419 completed the full posttest surveys. 209 were in the Web group, and 210 were in the print group. The characteristics of the participants are presented in Table 1.

**Table 1:** Demographic characteristics of field test participants

Characteristic	Web Group (N = 209)		Print Group (N = 210)	
	**n**	**%**	**n**	**%**
**Race**				
White	163	78.0	177	84.3
African American	18	8.6	11	5.2
Asian	12	5.7	9	4.3
Native Alaskan / Pacific Islander	2	1.0	1	0.5
Other	7	3.3	6	2.9
**Ethnicity**				
Hispanic	10	4.8	10	4.8
Non-Hispanic	184	88.0	191	91.0
**Gender**				
Male	53	25.4	62	29.5
Female	155	74.2	147	70.0
**Education**				
Less than high school	1	0.5	3	1.4
High school	7	3.3	8	3.8
Some college	70	33.5	71	33.8
Bachelor’s degree	79	37.8	78	37.1
Postgraduate degree	50	23.9	48	22.9
**Marital Status**				
Single	66	31.6	60	28.6
Married	125	59.8	28	61.0
Domestic partnership	10	4.8	9	4.3
Divorced	3	1.4	8	3.8
Widowed	2	1.0	1	0.5
Other	0	0.0	2	1.0
**Annual Income (US $)**				
20000-29999	0	0.0	3	1.4
30000-39999	13	6.2	18	8.6
40000-49999	32	15.3	19	9.0
Over 50000	143	68.4	154	73.3
**Age** (years), mean (range)	41.99 (24-64)		42.03 (22-66)	

### Measures

The measures contained in the online health surveys are listed below. The majority of these measures have been used in previously published studies by this research team and have shown evidence of reliability and validity in a variety of workplace settings and populations [7-11].

- Attitudes Toward a Healthful Diet: An 18-item scale assessing one’s attitudes toward healthful eating practices (alpha = .70). Higher score is better.
- Eating Practices: A 10-item scale assessing the frequency with which one engages in healthful eating practices (alpha = .63). Higher score is better.
- Motivation to Improve Diet: One item asking how important a healthy diet is to the respondent. Higher score is better.
- Dietary Behavioral Intentions: A 5-item scale assessing one’s intentions to eat a healthful diet (alpha = .86). Higher score is better.
- Dietary Self-Efficacy: A 6-item scale assessing one’s confidence in being able to eat a healthful diet (alpha = .89). Higher score is better.
- Dietary Stage of Change: A 4-item scale assessing one’s stage of change in adopting a healthful diet (alpha = .74). Lower score is better.
- Weight Stage of Change: A 4-item algorithm designed to classify one’s stage of change in losing weight or avoiding gaining weight [20]. Higher score is better.
- Perceived Stress: A 5-item scale assessing one’s level of perceived stress (alpha = .89). Lower score is better.
- Symptoms of Distress: A 15-item scale assessing one’s level of physical (alpha = .71) and emotional (alpha = .88) symptoms of stress. Lower score is better.
- Stress Stage of Change: One item assessing one’s stage of change in attempting to reduce stress. Lower score is better.
- Brief COPE: A 26-item measure, consisting of 13 two-item subscales, assessing one’s skills in coping with stress (alpha range: .47-.88) [21].
- Godin Leisure-Time Exercise Questionnaire: A 3-item scale assessing the frequency of physical activity in the past week (alpha = .78) [22]. Higher score is better.
- Godin Sweat Score: One item assessing one’s frequency of engaging in strenuous activity. Lower score is better.
- Exercise Stage of Change: One item assessing one’s stage of change in adhering to regular physical activity. Lower score is better
- Exercise Motivation: One item asking how important physical activity is to the respondent. Higher score is better.
- Exercise Behavioral Intentions: One item assessing one’s intentions to engage in regular physical activity. Higher score is better.
- Exercise Self-Efficacy: One item assessing one’s confidence in engaging in regular physical activity. Higher score is better.
- Weight: One item asking the respondent’s current weight.
- User Evaluations of Program (posttest-only): Five items in each of four health topics (stress, weight, diet/nutrition, and substance use) assessing the extent to which the materials (Web-based or print) provided information, helpful tips, motivation, good examples, and encouragement to examine practices. Lower score is better.
- Time and Frequency of Access (posttest Web group only): Respondent was asked to estimate frequency of and length of time (in minutes) spent accessing specific segments of the Web-based program.
- Ratings of Program Functions (posttest Web group only): A 10-item scale asking the respondent to rate the functionality (eg, ease of navigation, clarity of layout) of the program. Lower score is better.

A copy of all health outcome measures is available upon request.

### Equivalence of Groups

To determine the degree to which the randomization produced like samples, the experimental and control groups were compared on demographics and selected pretest dependent measures. To assess whether the experimental and control groups of the main study sample (those who completed both pretest and posttest) differed on selected dependent measures at pretest, analysis of variance (ANOVA) contrasted the two groups at pretest on five key dependent measures—Attitudes Toward a Healthful Diet, Eating Practices, Symptoms of Distress, Godin Sweat Score [22], and Weight. All comparisons were nonsignificant, indicating that the two groups were equivalent at pretest on the central outcomes of interest. Chi-square analyses contrasted the two groups on demographics at pretest—race, ethnicity, gender, education, marital status, and income—and found no significant differences, indicating that the two groups were demographically equivalent. The results of these analyses indicate that the randomization process was successful in producing equivalent groups.

ANOVA contrasting the pretest scores of the dropouts with those who completed both pretest and posttest on the same five dependent measures (Attitudes Toward a Healthful Diet, Eating Practices, Symptoms of Distress, Godin Sweat Score, and Weight) found no significant differences between the two groups, indicating that there was not differential attrition as a function of pretest position on key outcomes. Chi-square analyses contrasted the demographics (race, ethnicity, gender, education, marital status, and income) of the dropouts with those who completed both surveys and found no differences between the two groups, indicating that the demographic composition of the dropouts did not differ significantly from that of the participants who completed both surveys.

### Interventions

The Web-based program, Health Connection, is a comprehensive multimedia health promotion program offering substantial information and guidance on the major health promotion and wellness topics of stress management, nutrition/weight management, and fitness/physical activity. Conceptually rooted in accepted models of health behavior change as described above [12-13,15], the program was intended to be more than an information resource: it was designed to improve the health practices and attitudes of working adults in the three health topic areas. Many of the concepts and much of the content of the program were drawn from previous video-centered programs developed and tested by our group in a series of workplace-based studies [7-11].

The program was developed by the ISA Associates team over a 2-year period through multiple cycles of development and testing, beginning with focus group assessments of prototype content and culminating in the workplace-based field test. From the outset, the program was designed specifically for the broadband environment, anticipating that most workplaces would have DSL or higher Internet connections by 2005. Accordingly, the program, constructed in Macromedia Flash, is highly interactive with ample graphics, audio, and video (the entire program is audio-narrated). Many of these main elements are congruent with health behavior change theory and principles (eg, providing opportunities for observational learning, building self-efficacy, and self-tailoring of content and sequence). Sample screenshots are displayed in Figures 1a and 1b and Multimedia Appendix 1, and an outline of the program content is available in Multimedia Appendix 2.

The print materials consisted of five commercially available booklets:

1. “Low-Fat Eating” (15 pages)
2. “Getting Started with Weight Management” (15 pages)
3. “Stress Management: A Personal Action Guide” (15 pages)
4. “Fitness: The High Performance Lifestyle” (17 pages)
5. “Alcohol, Drugs, and a Healthy Lifestyle: What’s the Connection” (11 pages)

All booklets were colorful and included graphics and various tracking forms and logs. Outlines of the content of each booklet are presented in Multimedia Appendix 3.

**Figure 1a:**
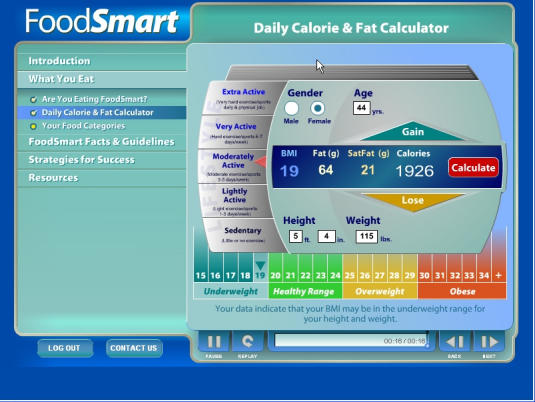
Health Connection screenshots

**Figure 1b:**
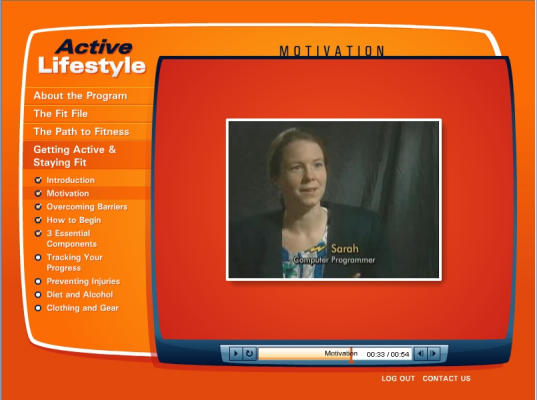
Health Connection screenshots

**Analysis**

Four major types of analyses were conducted on the health outcomes survey data: (1) analysis of covariance (ANCOVA), contrasting posttest measures of the Web and print groups with pretest measures as the covariate; (2) *t* tests of pretest-posttest changes within group; (3) dosage analyses using ANOVA to test for Web program exposure effects on dependent measures; and (4) ANOVA to compare user evaluations of the program (ratings of Web program vs print materials).

## Results

### Participation and Retention

There were approximately 5000 employees in the company, virtually all of whom were eligible to participate in the program. A total of 480 employees signed up for the program and participated in the pretest survey, roughly a 10% participation rate.

**Figure 2:**
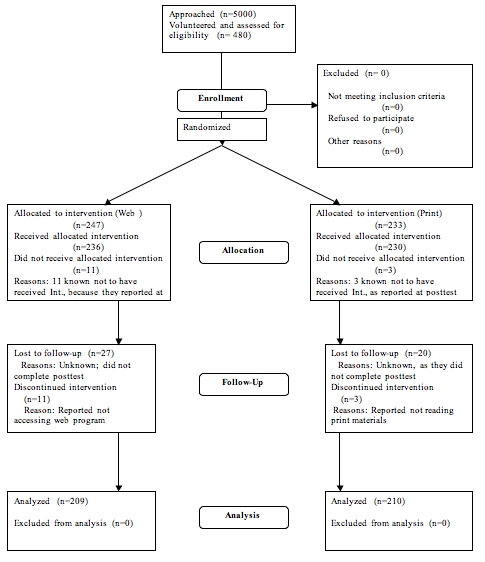
CONSORT Flowchart, displaying the flow of participants from enrollment through analysis.

There was a moderate amount of attrition from pretest to posttest, as 61 subjects — 38 from the Web group (85% retention rate) and 23 from the print group (87% retention rate) — who completed the pretest were not included in the final analysis, either because they did not complete the posttest or because they did not meet the criteria for inclusion. In the Web group, 27 subjects did not complete the posttest, and 11 subjects were excluded because they reported that they did not access the program. In the print group, 20 subjects did not complete the posttest, and 3 subjects claimed never to have received the print materials. The flow of participants through the study is shown in Figure 2.

For a more detailed analysis and discussion of the rates of participation and retention (along with frequency of Web program access), see Multimedia Appendix 4 for a PowerPoint presentation by Cook and associates.

### Eating Practices and Attitudes

Table 2 presents the results of the ANCOVA to test differential improvement on several measures of eating practices and attitudes. The Web group showed significantly greater improvement than the print group on measures of Attitudes Toward a Healthful Diet and Dietary Stage of Change. There were no other differences in the ANCOVA results between the two groups on these measures.

**Table 2:** Eating practices and attitudes

Measure	Web Posttest Mean (SD)	Print Posttest Mean (SD)	F (Degrees of Freedom)	*P*
Attitudes Toward a Healthful Diet^*^	3.835 (0.395)	3.711 (0.434)	7.104 (1,415)	.008
Eating Patterns^*^	2.695 (0.488)	2.641 (0.474)	0.004 (1,416)	.95
Dietary Motivation^*^	4.24 (0.786)	4.18 (0.850)	0.012 (1,416)	.91
Dietary Intentions^*^	4.126 (0.774)	4.189 (0.731)	0.198 (1,414)	.66
Dietary Self-Efficacy^*^	3.840 (0.759)	3.771 (0.784)	0.092 (1,414)	.76
Dietary Stage of Change^†^	1.902 (0.902)	2.149 (0.921)	6.487 (1,408)	.01
Weight Stage of Change^*^	3.00 (0.909)	3.00 (0.712)	0.286 (1,403)	.59

^*^Higher score is better.

^†^Lower score is better.

Table 3 shows the results of tests of pretest-posttest changes in dietary outcomes for the Web group, showing that the Web group produced significant pretest-posttest improvements on all seven outcome measures. However, the print group showed similar significant improvements, with the exception of a nonsignificant change in Weight Stage of Change.

**Table 3:** Pretest-posttest changes: eating practices and attitudes

Measure	Pretest Mean (SD)	Posttest Mean (SD)	*t*	*P*
Attitudes Toward a Healthful Diet^*^				
Web	3.6358 (0.4309)	3.8354 (0.3950)	−7.673	< .001
Print	3.5698 (0.4586)	3.7113 (0.4344)	−5.051	< .001
Eating Patterns^*^				
Web	2.5929 (0.50295)	2.6949 (0.4884)	−4.717	< .001
Print	2.5256 (0.4669)	2.6408 (0.4743)	−5.174	< .001
Dietary Motivation^*^				
Web	4.24 (0.786)	4.13 (0.783)	2.506	.01
Print	4.18 (0.850)	4.04 (0.797)	2.771	.01
Dietary Intentions^*^				
Web	3.9116 (0.8713)	4.1256 (0.7736)	−4.147	< .001
Print	3.984 (0.7979)	4.1895 (0.7313)	−3.972	< .001
Dietary Self-Efficacy^*^				
Web	3.5533 (0.8207)	3.8403 (0.7587)	−5.494	< .001
Print	3.4562 (0.8503)	3.7711 (0.7844)	−5.978	< .001
Dietary Stage of Change^†^				
Web	2.306 (1.061)	1.902 (0.902)	6.167	< .001
Print	2.4199 (0.9989)	2.1486 (0.9214)	4.385	< .001
Weight Stage of Change^*^				
Web	2.84 (0.880)	3.00 (0.909)	−2.747	.01
Print	2.92 (0.735)	3.00 (0.712)	−1.694	.09

^*^Higher score is better.

^†^Lower score is better.

### Weight

The ANCOVA analyses for reported weight indicated that there was no significant differential change in weight between the two groups. However, both groups achieved a significant, though small, reduction in reported weight from pretest to posttest. The Web group lost an average of 0.57 kg (*t* = 2.09, *P* = .04), and the print group lost an average of 0.96 kg (*t* = 2.33, *P* = .02).

### Stress Management

The ANCOVA to test differential change between the Web group and the print group detected no differences between the two groups on the three measures of stress. In addition, none of the 13 subscales of Coping Skills (2-item subscales, scored individually, equaling 26 items) yielded differences between the groups.

Table 4 displays the results of tests of pretest-posttest change in stress outcomes, showing significant improvement by the Web group on Symptoms of Distress; however, the print group showed significant pretest-posttest improvement on all three stress outcome measures.

**Table 4:** Pretest-posttest changes: stress management

Measure^*^	Pretest Mean (SD)	Posttest Mean (SD)	*t*	*P*
Perceived Stress				
Web	14.21 (4.936)	13.6746 (4.8773)	1.780	.08
Print	15.05 (4.645)	14.2714 (4.545)	2.862	.01
Stress Stage of Change				
Web	2.40 (1.555)	2.30 (1.504)	0.915	.36
Print	2.53 (1.535)	2.15 (1.417)	3.716	< .001
Symptoms of Distress				
Web	30.29 (8.22)	29.14 (8.386)	2.474	.01
Print	31.62 (7.922)	30.03 (7.659)	3.813	< .001

^*^Lower score is better.

### Physical Activity

The ANCOVA to test differential change between the Web group and the print group detected no differences between the two groups on the five measures of physical activity. As shown in Table 5, the tests of pretest-posttest changes within groups showed that both groups achieved significant improvement on the Godin Sweat Score [22] and the Activity Stage of Change measure, while the print group also showed significant improvement on the Activity Confidence measure (although Web group came close).

**Table 5:** Pretest-posttest changes: physical activity

Measure	Pretest Mean (SD)	Posttest Mean (SD)	*t*	*P*
Godin Leisure Score^*^				
Web	46.55 (55.866)	57.35 (121.588)	−1.688	.09
Print	42.81 (62.115)	46.53 (35.916)	−0.802	.42
Godin Sweat Score^†^				
Web	2.08 (0.751)	1.91 (0.646)	3.454	.001
Print	2.17 (0.784)	1.94 (0.705)	4.546	< .001
Activity Stage of Change^†^				
Web	2.26 (1.286)	1.97 (1.191)	3.682	< .001
Print	2.50 (1.279)	2.13 (1.192)	4.556	< .001
Activity Motivation^*^				
Web	4.12 (0.953)	4.06 (1.052)	1.051	.29
Print	4.14 (0.898)	4.11 (1.098)	0.573	.57
Activity Intentions^*^				
Web	4.53 (0.791)	4.60 (0.694)	−1.414	.16
Print	4.48 (0.816)	4.56 (0.778)	−1.490	.14
Activity Confidence^*^				
Web	3.80 (1.194)	3.94 (1.020)	−1.896	.06
Print	3.51 (1.217)	3.81 (1.131)	−4.068	< .001

^*^Higher score is better.

^†^Lower score is better.

### Dosage Analysis

In order to assess the extent to which the Web program effects were a function of the number of times the user accessed a particular program module (stress management, physical activity, and nutrition/weight control), a series of dosage analyses were conducted on the outcome measures. The Web group participants were classified into three groups: those who reported that they never accessed the particular module, those who reported accessing it once, and those who reported accessing it more than once. The analyses tested for (1) linear trends by group and (2) multiple contrasts by group for all outcome measures. The multiple contrasts also included the print group as a fourth group.

The results of the trend analysis on dietary measures, shown in Table 6, indicate that there were significant linear effects of the Web-based nutrition/weight control module on three of the seven dietary measures: Self-Efficacy, Attitudes Toward a Healthful Diet, and Dietary Stage of Change.

**Table 6:** Nutrition/weight control module effects by dosage: trend analyses

	Mean Score by Number of Times Accessed (No.)			Overall		Linear
Measure	Never	Once	More Than Once	F (Degrees of Freedom)	*P*	*P*
Dietary Self-Efficacy	3.579 (28)	3.781 (93)	3.989 (86)	5.270 (2,203)	.006	.003
Attitudes Towards a Healthful Diet	3.751 (28)	3.809 (93)	3.890 (87)	2.585 (2,204)	.08	.04
Eating Patterns	2.678 (28)	2.695 (94)	2.700 (87)	.059 (2,205)	.94	.73
Dietary Motivation	4.090 (28)	4.229 (94)	4.310 (87)	1.473 (2,205)	.23	.09
Dietary Intentions	4.110 (27)	4.075 (93)	4.184 (87)	.694 (2,203)	.50	.59
Dietary Stage of Change	2.199 (28)	1.961 (92)	1.738 (84)	4.627 (2,200)	.01	.01
Weight Stage of Change	3.118 (27)	2.894 (92)	3.079 (83)	1.682 (2,198)	.19	.82

The analysis of contrasts of the print group (who never accessed the nutrition/weight control module) with the three different levels of access of the nutrition/weight control module in the Web group also produced significant dosage effects for the same three dietary measures. On all three measures, the significant contrast occurred in the print group versus the Web group that accessed the module more than once (Table 7).

**Table 7:** Nutrition/weight control module effects by dosage: multiple contrasts

	Contrasts of the Print Group vs Levels of Web Access, *P* values			Overall Contrast	
Measure	Never	Once	More Than Once	F (Degrees of Freedom)	*P*
Dietary Self-Efficacy	.06	.61	.04	3.422 (3,412)	.02
Attitudes Towards a Healthful Diet	.96	.14	.001	3.937 (3,413)	.01
Eating Patterns	.80	.95	.86	0.039 (3,414)	.99
Dietary Motivation	.25	.92	.38	0.883 (3,414)	.45
Dietary Intentions	.74	.31	.69	0.542 (3,412)	.65
Dietary Stage of Change	.48	.17	.00	5.218 (3,406)	.002
Weight Stage of Change	.31	.42	.18	1.457 (3,401)	.23

The findings from the dosage analysis are similar to those for the two-group Web versus print ANCOVA, except that the dosage analysis—both trend and multiple contrasts—revealed significant effects of the Web program on the measure of Dietary Self-Efficacy as well as Attitudes Toward a Healthful Diet, and Dietary Stage of Change.

The dosage analyses conducted for the other two modules (physical activity and stress management) found no significant dosage effects for either.

### Program Evaluations

At posttest, all subjects rated the program materials, either the Web-based program or the print materials, using a 5-point scale. In each of three topic areas—stress, diet, and weight management—subjects rated the materials on the degree to which they (1) “provided a wealth of information,” (2) “gave me helpful tips,” (3) “motivated me,” (4) provided good examples,” and (5) “encouraged me to examine [the topic].” Within each topic area, the ratings were averaged and ANOVA was used to test differences between groups on these average ratings. As shown in the first three rows of Table 8, the Web group ratings significantly exceeded the print group in all three topic areas.

Subjects also rated the extent which the materials (Web-based or print) were “engaging and appealing,” and “easy to access and understand.” The last three rows of Table 8 show that the ratings for the Web-based program significantly exceeded the print materials for the first measure and in the average of the two ratings (overall). Although the Web-based program received higher ratings for the second measure, the difference was not statistically significant.

**Table 8:** Program evaluations

Segment/Topic^*^	Web Posttest Mean (SD)	Print Posttest Mean (SD)	F (Degrees of Freedom)	*P*
Stress management	2.00 (0.73)	2.15 (0.61)	4.748 (1,395)	.03
Diet/nutrition	1.86 (0.67)	2.08 (0.67)	11.380 (1,398)	.001
Weight management	1.99 (.073)	2.16 (0.69)	5.721 (1,392)	.02
				
Engaging/appealing	1.79 (0.86)	2.09 (0.87)	12.156 (1,410)	.001
Accessible/ understandable	1.50 (0.55)	1.60 (0.50)	3.233 (1,409)	.07
Overall evaluation	1.65 (0.64)	1.84 (0.60)	9.808 (1,410)	.002

^*^Lower score is better.

## Discussion

### Review and Interpretation of Findings

The direct comparisons of the Web group and print group showed that the Web-based program performed significantly better than the print materials on measures of Attitudes Toward a Healthful Diet and Dietary Stage of Change, and the dosage analysis indicated significant effects of the Web-based nutrition/weight management segment on Dietary Self-Efficacy as well. In addition, the Web-based program performed significantly better than the print materials on virtually all dimensions of program evaluation. However, on outcome measures of stress and physical activity, there were no significant differences between those who received the Web-based program and those who received the print materials.

Of particular interest was the finding that both the Web group and the print group showed significant improvements on multiple measures in each topic area (diet, stress, etc) from pretest to posttest, suggesting that either (1) both interventions were effective in improving health practices and attitudes or (2) the improvements were the spurious result of some other force or event, perhaps an overall social desirability effect, a Hawthorne effect, or a function of most subjects in both groups coming to the field test with substantial motivations to improve their health. Because it was originally thought that the print materials would form a relatively weak intervention compared to the Web program, a no-treatment control was not included in the design; thus, it is difficult to determine which interpretation is the correct one. It is possible that the improvements occurred as a result of a secular or environmental change affecting both groups, such as an easing of the workload across the workforce (unlikely) or the implementation of another health promotion program in all three offices (this was not the case). It is also possible that both groups exhibited a quasi-placebo effect: wanting to improve their health practices and given some impetus to do so (the surveys and programs), they reported improvements across most of the measures, but the improvements were not a function of the interventions. However, the fact that the improvements occurred consistently in the predicted direction across the majority of these measures, including reductions in reported weight from pretest to posttest, argues against this interpretation.

Perhaps the most plausible explanation is that most of the participants in both groups entered the study with considerable motivation to make improvements in their health practices and attitudes, and both the Web-based program and the print materials contained sufficient information and guidance to help participants make the desired improvements. Indeed, for both groups, the pretest motivation items for improving dietary practices and for increasing activity exceeded 4.0 on a 5-point scale, indicating that both groups were indeed motivated to make positive changes in diet and activity upon entering the field test. Although the print group was originally conceived as a control group, it seems likely that the scope and quality of the print materials—five colorful booklets that included tracking forms and logs, totaling 73 pages—were such that the print materials were effective interventions in themselves and constituted a substantial rival to the Web program. As noted earlier, the effectiveness of print materials for changing health behaviors and outcomes is supported in the literature [16-19]. In comparison to the print materials, the Web program contained more total content and was presented in a multimedia format; hence, the Web program received higher ratings for being appealing and motivating, providing a wealth of information, and for other evaluation measures than the print materials. Indeed, the superiority of the Web program over the print materials in the participant ratings was striking: across all topic areas, the Web group ratings significantly exceeded the print ratings on 15 items; the print group ratings exceeded the Web group on none. The Web group program consistently garnered higher ratings for “wealth of information,” “helpful tips,” “motivated me,” “provided good examples,” and “encouraged me to examine [the topic].”

On the other hand, the print materials offered other advantages: users had all the material in hand from the start, with no need to access the Web or navigate the program, and they could carry one or more of the booklets virtually anywhere they went. The apparent efficacy of the print materials suggests that increasing the number of printable downloads in the Web program would improve its effectiveness.

The findings from the dosage analysis further support the view that the effects of the Web program were real and not a result of social desirability or other nonprogram effects, at least in the dietary topic area. On multiple measures, the Web program effects were a positive linear function of the number of times users accessed the program: as exposure to the program increased, so did the size of the improvements. As noted in the presentation by Cook et al (see Multimedia Appendix 4), the frequency with which users accessed the Web-based program was lower than anticipated and was especially low for the modules on stress and physical activity, a finding which suggests, along with the dosage analysis, that frequency of access might be related to program effects. Although the causal direction is unclear, taken together these findings suggest that procedures designed to increase the frequency of user access (eg, tying incentives to frequency of access) might improve both adherence and efficacy.

### Related Studies

The results of this study are generally congruent with findings from other evaluations of Web-based health interventions, although this Web-based program addressed a wider variety of health topics than most previous interventions, which have typically focused on a single health topic. The meta-analysis of Web-based versus non-Web-based interventions conducted by Wantland and associates concluded that effect size comparisons across the 22 studies showed improvement in knowledge and/or behavior change health outcomes for individuals using Web-based interventions [5]. However, a closer examination of the three studies in the meta-analysis that focused on nutrition/weight management and physical activity reveals that the results for these particular studies were less uniformly positive than the overall conclusions seemed to indicate. The assessment of a Web-based intervention to facilitate weight loss found a modest effect size of .15, and the authors concluded that the Web-based intervention was not as effective as in-person support [23]. A randomized control trial of the efficacy of a Web-based tailored nutrition education program found the Web-based group to be significantly better than the control group on posttest measures of nutrition awareness and intentions to change, concluding that Web-based interventions can lead to changes in *determinants* of behavior [24]. A study comparing Web-based and print versions of a work site physical activity program showed no significant increase in physical activity in either group, although the Web group showed a significant decrease in time reported sitting [25].

However, although the studies of Web-based weight management programs reviewed by Wantland et al did not generate evidence of strong effects, recent evaluations of other Web-based weight management programs [26-28] have produced positive findings, indicating that the positive results on dietary measures found in this study are congruent with other, perhaps similar Web-based programs. In particular, with its emphasis on healthy eating, the relationship between food consumption and energy expenditure, tips on overcoming barriers, and so on, the Balance program studied by Rothert and associates [28] appears to be quite similar to the Health Connection program, although more structured and focused specifically on weight loss among those with a body mass index above 27. Their program showed substantial effects in the dietary area, as participants in the Balance program lost significantly more weight than participants in an information-only condition, and more Balance participants than information-only participants reported that the program was relevant, helpful, and easy to understand. However, attrition in the Rothert study was much greater than in the present study, as only 30% and 20% of the baseline sample responded to the follow-up surveys at 3 and 6 months, respectively—a significant limitation of the study, as noted by the authors. The much higher retention rate in the present study was probably due in part to the monetary incentives tied to survey responses, while the Rothert study offered no incentives.

In some contrast to the relatively positive findings from this study and others on Web-based dietary and weight management programs, studies of Web-based programs aimed at stress management and increasing physical activity are less numerous and, as indicated by the Wantland review [5], somewhat less efficacious. The relative paucity of Web-based programs aimed at stress and activity compared to weight management is doubtless a reflection of the interests of the general population and the increasing importance placed on weight control by medical professionals. However, the lesser degree of efficacy found in this study as well as others on levels of stress and activity suggests the possibility that these topics might be less suited to Web-based approaches than programs aimed at weight control, or that Web-based techniques in the areas of stress and activity programs are less advanced than in the dietary area.

It should be emphasized that Web-based programs vary widely not only in content but also in the degree to which they are science-based (both scientifically accurate and rooted in accepted theories of health behavior change), media-rich, and interactive. Web-based programs containing questionable information or lacking accepted theoretical foundations are unlikely to be effective [28,29].

There are substantial differences in the level of effort and costs of implementing and distributing Web- and print-based approaches. Providing access to the Web program required only a single email message sent instantaneously to all participants, whereas the print materials had to be mailed individually (through company mail) to all participants located in three different offices across the United States. Providing a Web-based program to users is also probably less costly than providing print materials, except perhaps for very small workforces. Indeed, once built, Web-based programs can be delivered to millions of users in a very cost-effective manner, reaching audience sizes unattainable by the traditional workplace health promotion programs.

### Limitations

Limitations of the study include the following: (1) all dependent measures were self-reports; (2) the test period was limited to three months; and (3) a no-treatment control condition was not included. Future research on the efficacy of Web-based health promotion interventions should include the collection of physical/biological measures and health care utilization data, should assess participants for longer periods or time, and should include a no-treatment control group.

## Multimedia Appendix 1

Selected screenshots of the Health Connection program (pdf)
											

## Multimedia Appendix 2

Outline of Health Connection Web program content (pdf)
											

## Multimedia Appendix 3

Detailed outline of print materials reviewed by the control group (pdf)
											

## Multimedia Appendix 4

Presentation on adherence in Internet interventions aimed at diet, exercise, and stress presented at the 11th World Congress on Internet in Medicine (http://www.mednetcongress.org)  (ppt)
											

## References

[1] Pelletier Kenneth R (2005). A review and analysis of the clinical and cost-effectiveness studies of comprehensive health promotion and disease management programs at the worksite: update VI 2000-2004. J Occup Environ Med.

[2] Ozminkowski Ronald J, Ling Davina, Goetzel Ron Z, Bruno Jennifer A, Rutter Kathleen R, Isaac Fikry, Wang Sara (2002). Long-term impact of Johnson & Johnson's Health & Wellness Program on health care utilization and expenditures. J Occup Environ Med.

[3] Goetzel Ron Z, Ozminkowski Ronald J, Bruno Jennifer A, Rutter Kathleen R, Isaac Fikry, Wang Shaohung (2002). The long-term impact of Johnson & Johnson's Health & Wellness Program on employee health risks. J Occup Environ Med.

[4] Evers Kerry E (2006). eHealth promotion: the use of the Internet for health promotion. Am J Health Promot.

[5] Wantland Dean J, Portillo Carmen J, Holzemer William L, Slaughter Rob, Mcghee Eva M (2004). The effectiveness of Web-based vs. non-Web-based interventions: a meta-analysis of behavioral change outcomes. J Med Internet Res.

[6] McPherson Tracy L, Cook Royer F, Back Anita S, Hersch Rebekah K, Hendrickson April (2006). A field test of a Web-based substance abuse prevention training program for health promotion professionals. Am J Health Promot.

[7] Cook RF, Back A, Trudeau J (1996). Substance abuse prevention in the workplace: recent findings and an expanded conceptual model. J Prim Prev.

[8] Cook R F, Back A S, Trudeau J (1996). Preventing alcohol use problems among blue-collar workers: a field test of the Working People program. Subst Use Misuse.

[9] Cook RF, Back AS, Trudeau JV, McPherson TL (2003). Integrating substance abuse prevention into health promotion programs in the workplace: a social cognitive intervention targeting the mainstream user. Bennett JB, Lehman WEK, editors. Beyond Drug Testing: Innovative Approaches to Dealing with Employee Substance Abuse.

[10] Cook RF, Hersch RK, Back AS, McPherson TL (2004). The prevention of substance abuse among construction workers: a field test of a social cognitive program. J Prim Prev.

[11] Deitz Diane, Cook Royer, Hersch Rebekah (2005). Workplace health promotion and utilization of health services: follow-up data findings. J Behav Health Serv Res.

[12] Bandura A (1977). Social Learning Theory.

[13] Bandura A (1986). Social Foundations of Thought and Action: A Social Cognitive Theory.

[14] Cook R F, Youngblood A (1990). Preventing substance abuse as an integral part of worksite health promotion. Occup Med.

[15] Prochaska J O, DiClemente C C, Norcross J C (1992). In search of how people change. Applications to addictive behaviors. Am Psychol.

[16] Humpel N, Marshall A L, Iverson D, Leslie E, Owen N (2004). Trial of print and telephone delivered interventions to influence walking. Prev Med.

[17] Marshall Alison L, Bauman Adrian E, Owen Neville, Booth Michael L, Crawford David, Marcus Bess H (2003). Population-based randomized controlled trial of a stage-targeted physical activity intervention. Ann Behav Med.

[18] Apodaca Timothy R, Miller William R (2003). A meta-analysis of the effectiveness of bibliotherapy for alcohol problems. J Clin Psychol.

[19] Den Boer P C, Wiersma D, Van Den Bosch R J (2004). Why is self-help neglected in the treatment of emotional disorders? A meta-analysis. Psychol Med.

[20] Weight: Stages of Change - Short Form. Cancer Prevention Research Center.

[21] Carver C S (1997). You want to measure coping but your protocol's too long: consider the brief COPE. Int J Behav Med.

[22] Godin G, Shephard R J (1985). A simple method to assess exercise behavior in the community. Can J Appl Sport Sci.

[23] Harvey-Berino J, Pintauro S, Buzzell P, DiGiulio M, Casey Gold B, Moldovan C, Ramirez E (2002). Does using the Internet facilitate the maintenance of weight loss?. Int J Obes Relat Metab Disord.

[24] Oenema A, Brug J, Lechner L (2001). Web-based tailored nutrition education: results of a randomized controlled trial. Health Educ Res.

[25] Marshall Alison L, Leslie Eva R, Bauman Adrian E, Marcus Bess H, Owen Neville (2003). Print versus website physical activity programs: a randomized trial. Am J Prev Med.

[26] Tate Deborah F, Jackvony Elizabeth H, Wing Rena R (2006). A randomized trial comparing human e-mail counseling, computer-automated tailored counseling, and no counseling in an Internet weight loss program. Arch Intern Med.

[27] Tate Deborah F, Jackvony Elizabeth H, Wing Rena R (2003). Effects of Internet behavioral counseling on weight loss in adults at risk for type 2 diabetes: a randomized trial. JAMA.

[28] Rothert Kendra, Strecher Victor J, Doyle Laurie A, Caplan William M, Joyce Jodi S, Jimison Holly B, Karm Lya M, Mims Adrienne D, Roth Mark A (2006). Web-based weight management programs in an integrated health care setting: a randomized, controlled trial. Obesity (Silver Spring).

[29] Womble Leslie G, Wadden Thomas A, McGuckin Brian G, Sargent Stephanie L, Rothman Rebecca A, Krauthamer-Ewing E Stephanie (2004). A randomized controlled trial of a commercial internet weight loss program. Obes Res.

