# Supplementary material for: A Field Test of a Web-Based Workplace Health Promotion Program to Improve Dietary Practices, Reduce Stress, and Increase Physical Activity: Randomized Controlled Trial
Source: J Med Internet Res. 2007 Jun 19;9(2):e17. doi: 10.2196/jmir.9.2.e17 (PMC1913939; doi:10.2196/jmir.9.2.e17)

Health Connection: Stress Management

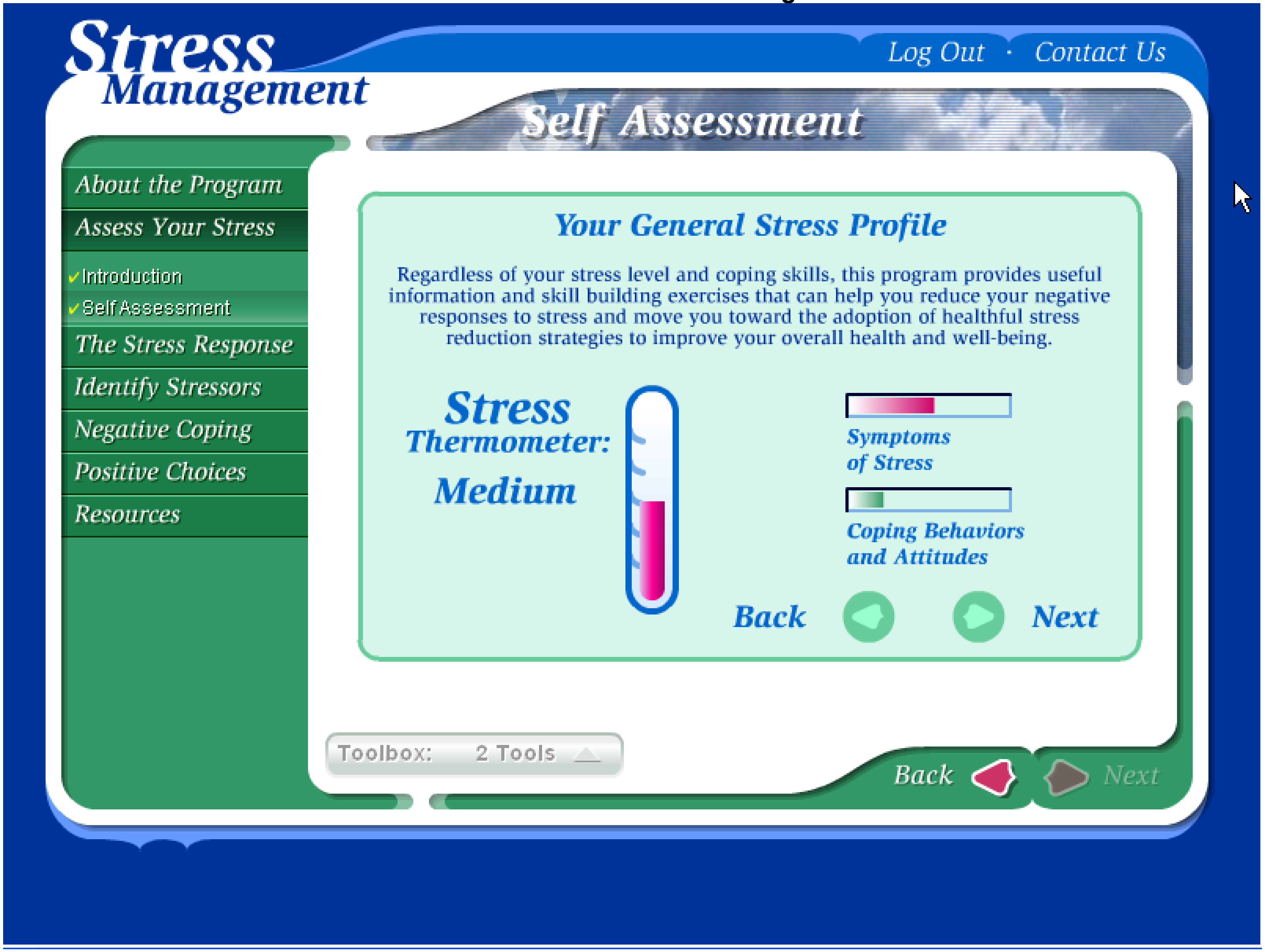

Health Connection: Stress Management

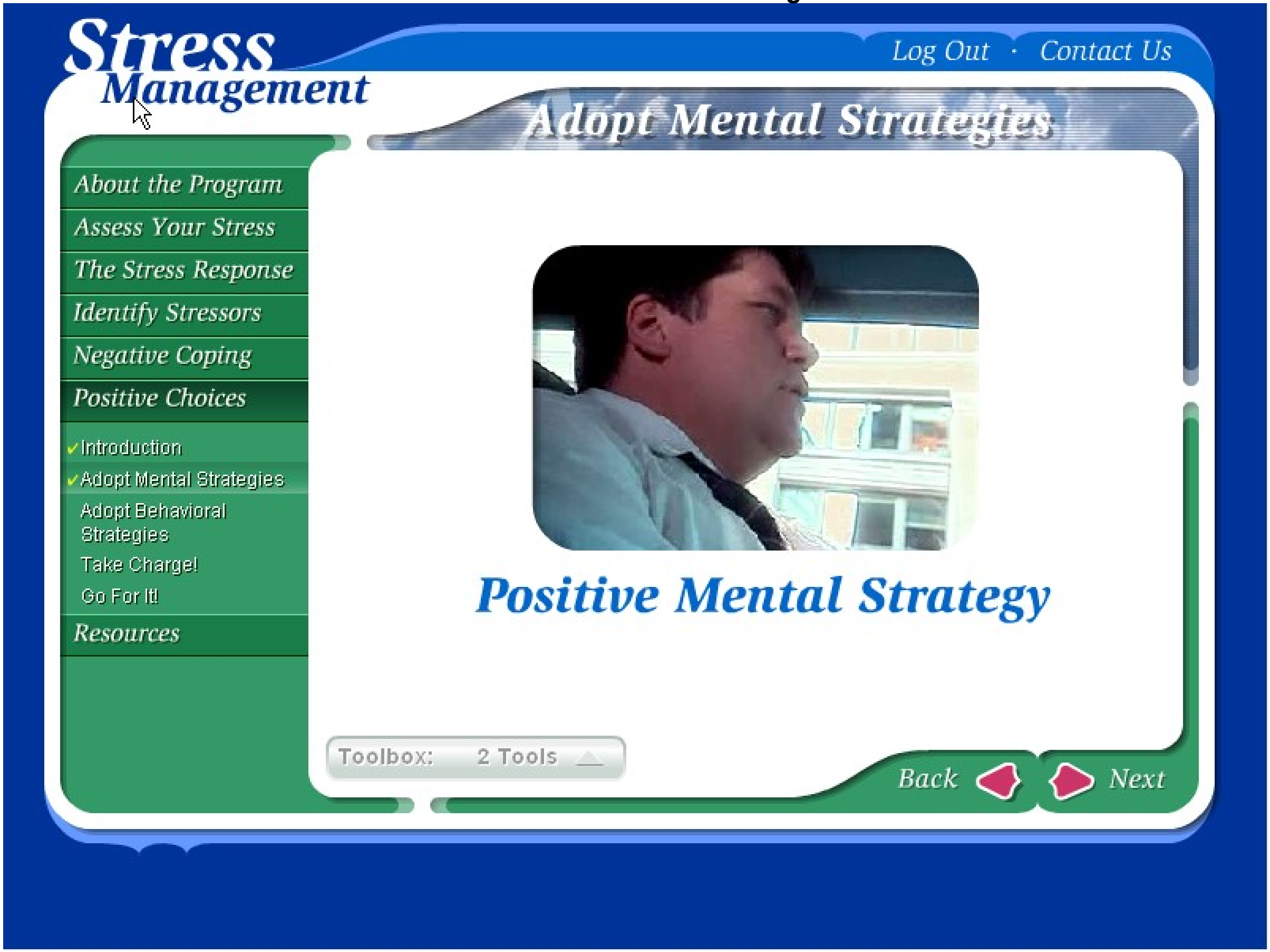

Health Connection: Active Lifestyle

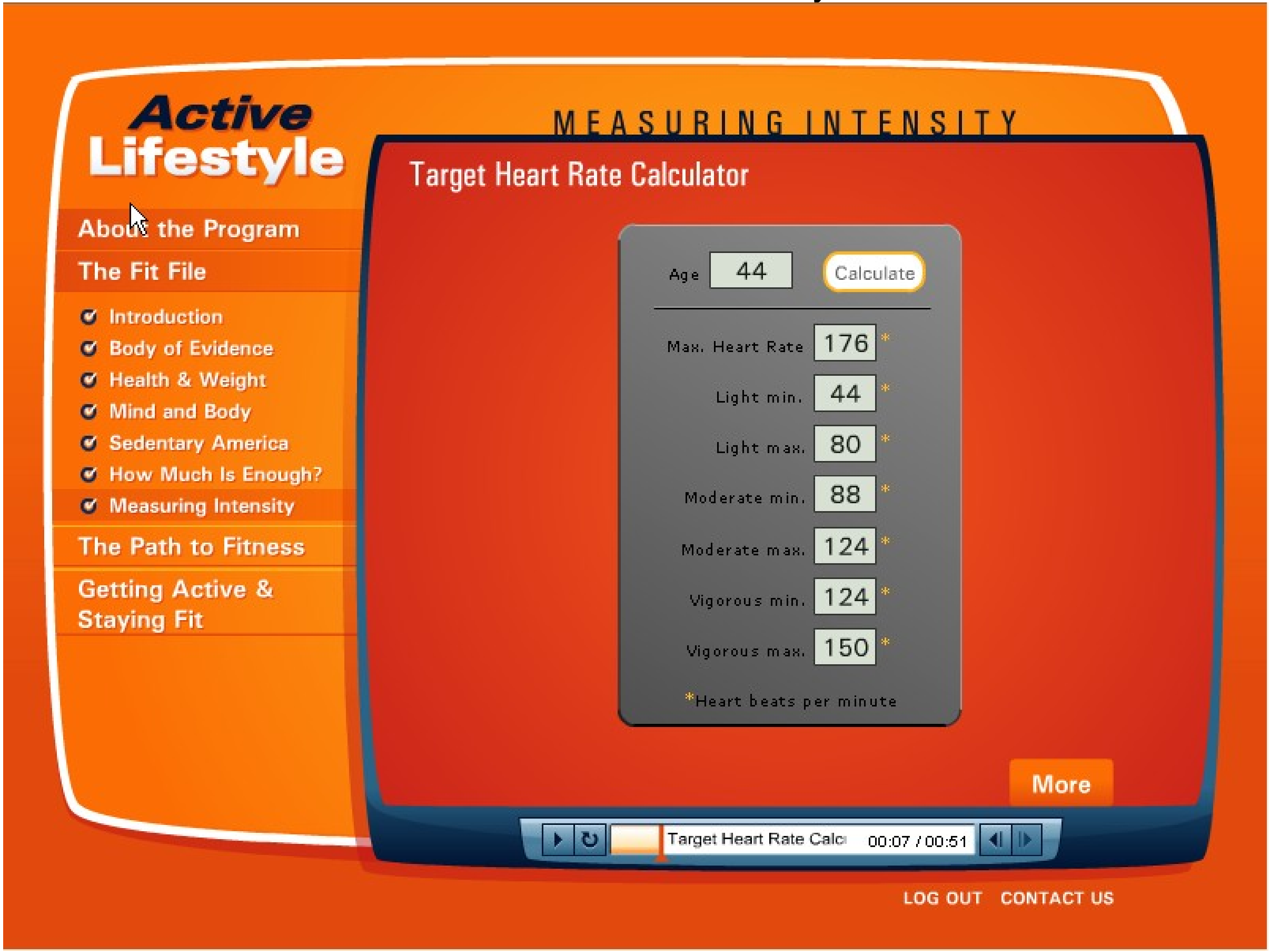

Health Connection: Active Lifestyle

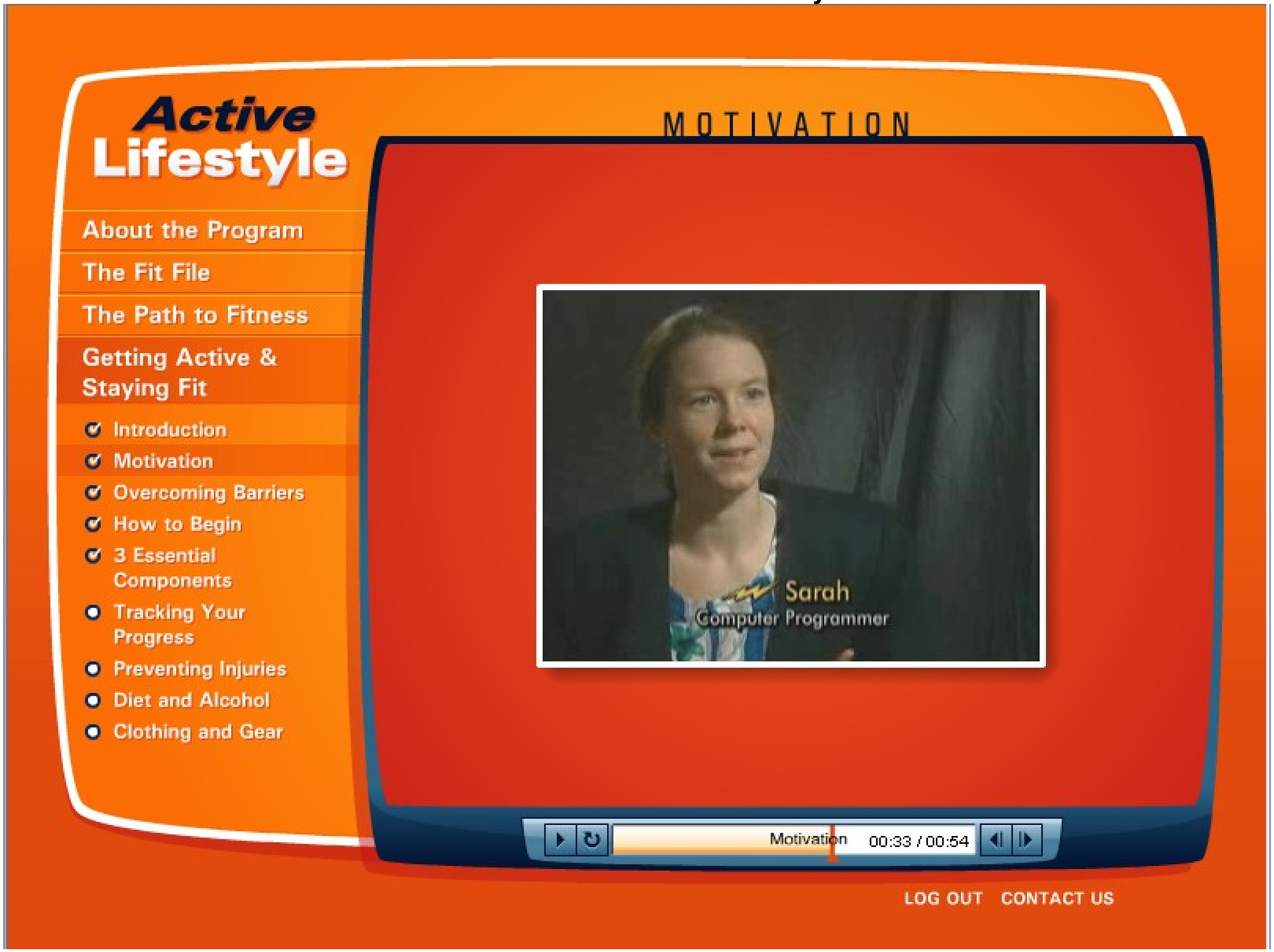

Health Connection: Food Smart

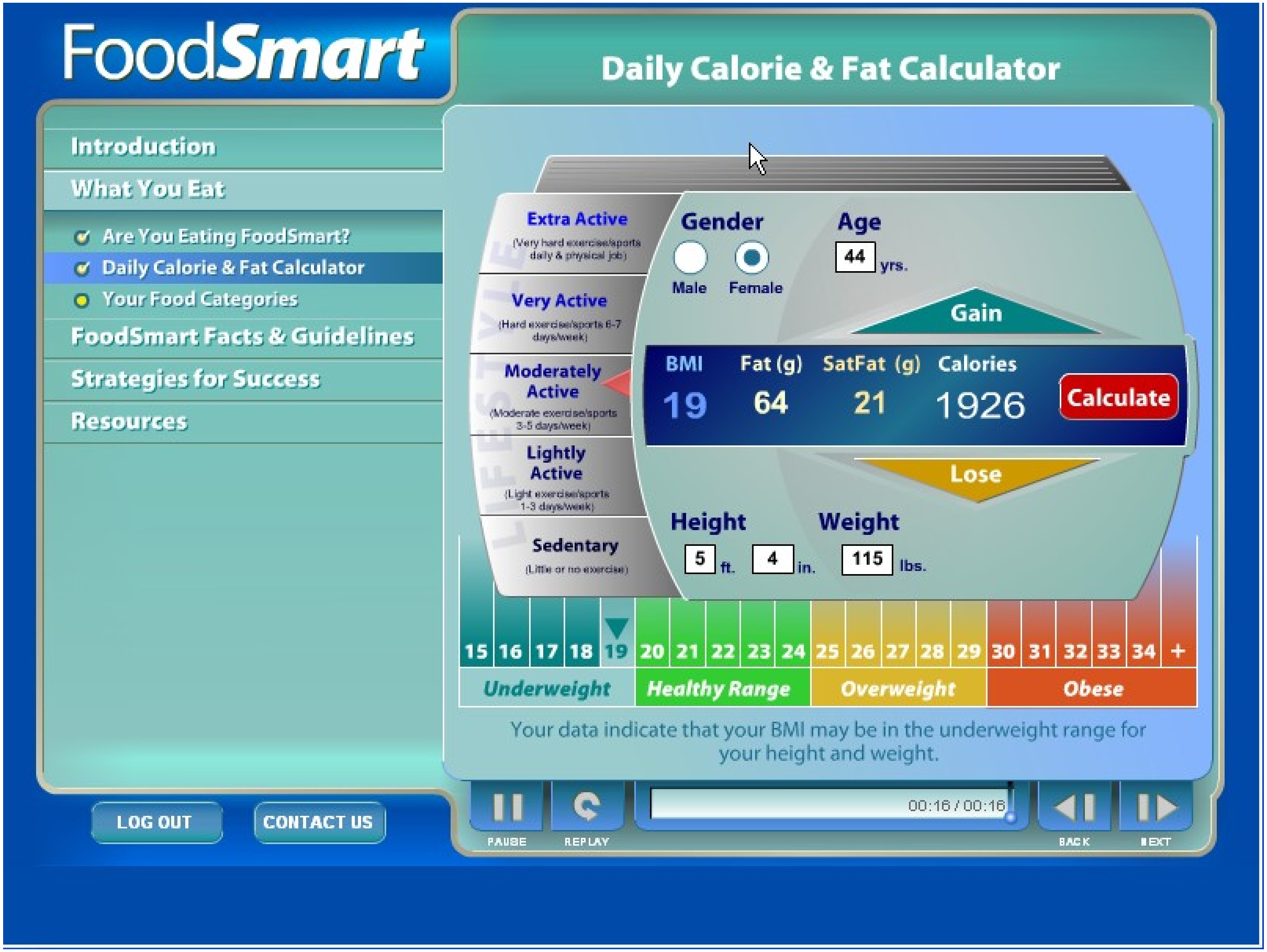

Health Connection: Food Smart

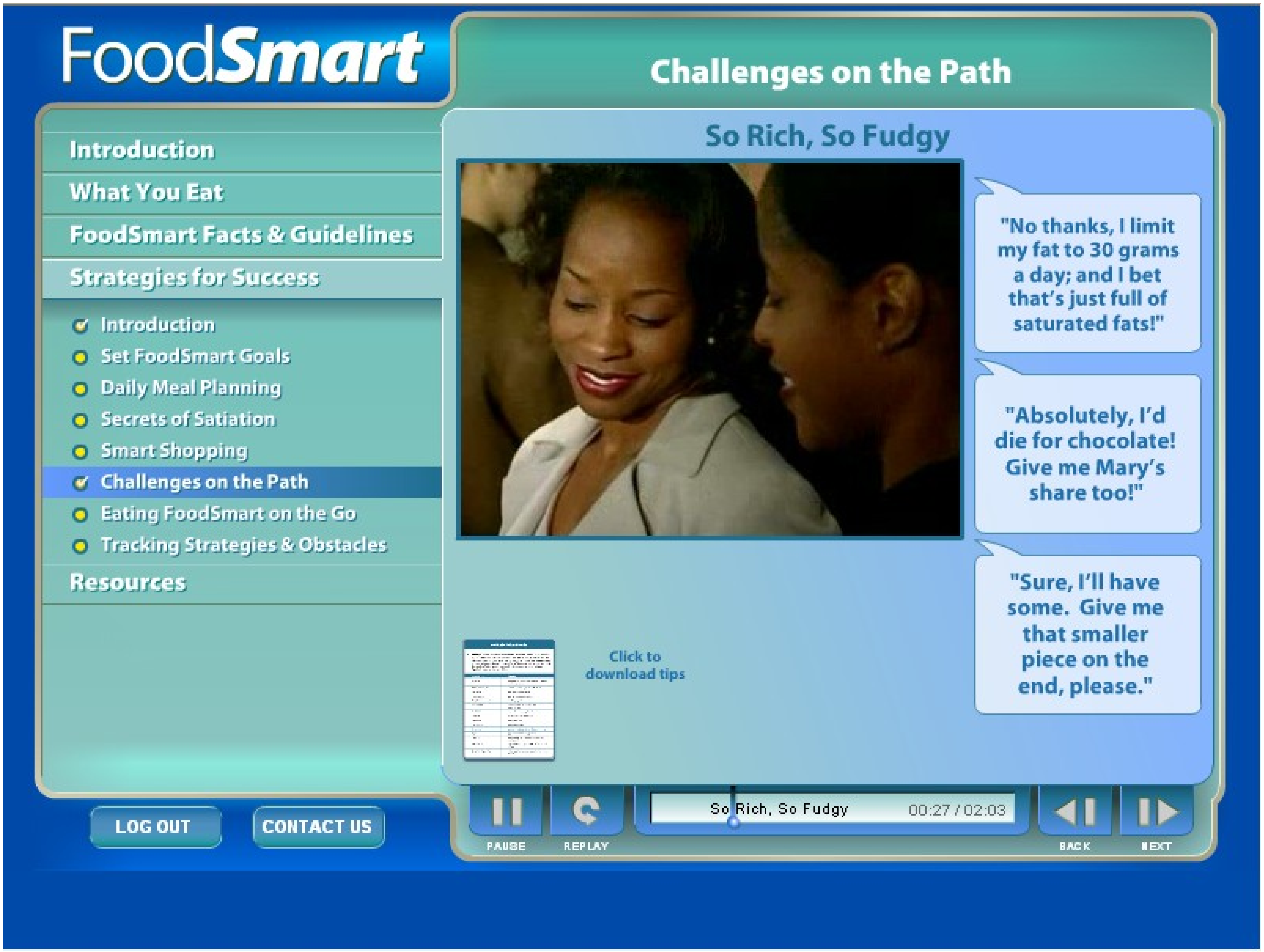

Health Connection: Food Smart

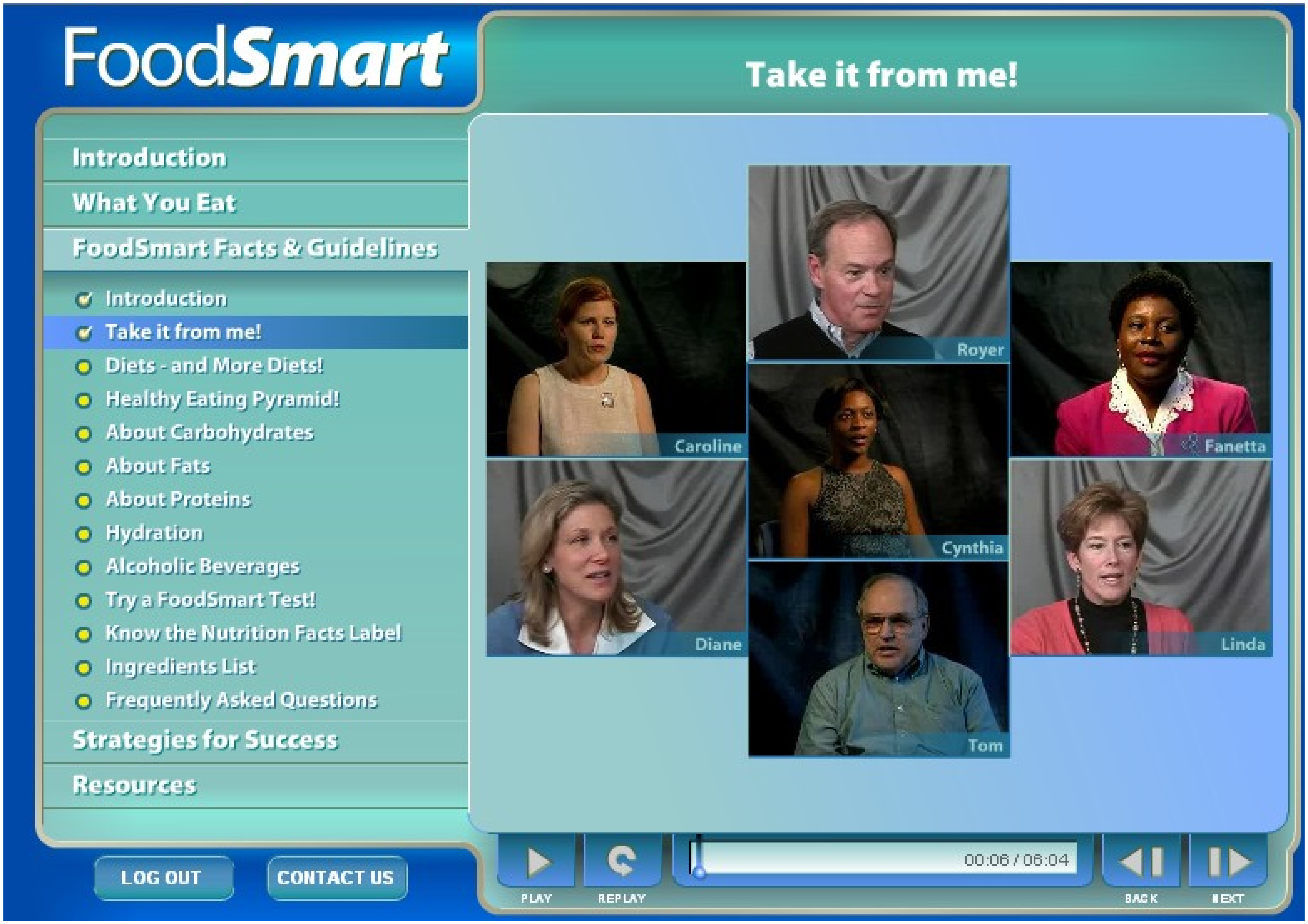

Supplement: Supplementary file 1 [file jmir_v9i2e17_app1.pdf]
