# Supplementary material for: A Field Test of a Web-Based Workplace Health Promotion Program to Improve Dietary Practices, Reduce Stress, and Increase Physical Activity: Randomized Controlled Trial
Source: J Med Internet Res. 2007 Jun 19;9(2):e17. doi: 10.2196/jmir.9.2.e17 (PMC1913939; doi:10.2196/jmir.9.2.e17)
Supplement: Supplementary file 2 [file jmir_v9i2e17_app2.pdf]

Cook RF, Billings DW, Hersch RK, Back AS, Hendrickson A
A Field Test of a Web-Based Workplace Health Promotion Program to Improve Dietary Practices, Reduce Stress, and Increase Physical Activity: Randomized Controlled Trial
J Med Internet Res 2007;9(2):e17
<URL: http://www.jmir.org/2007/2/e17/>

Multimedia Appendix 2

Health Connection
Web-Based Program: Outline of Content

Health Connection
Web-Based Program: Outline of Content

*Active Lifestyle (*Physical Activity*)*

The Fit File

- Information about the difference between exercise and physical activity
- Education about the benefits of moderate physical activity including
  - Preventing the occurrence of chronic physical illness
  - Promoting mental health
  - Reducing or maintaining a healthy body weight
- Instruction in three different methods to determine the optimal intensity of physical activity for health and well-being benefits
  - Including an interactive calculator to determine target heart rate

The Path to Fitness

- Self-assessment of readiness to incorporate physical activity into daily life. Based on response, personalized strategies include:
  - Interactive exercise examining the costs and benefits of physical activity.
  - Setting SMART (specific, measurable, achievable, rewarding, and timed) goals
  - Ways to find the time for physical activity
  - Relying on social support
  - Monitoring activity
  - Anticipating setbacks
  - Rewarding efforts
  - Varying routine
  - Mentoring others

Getting Active and Staying Fit

- Education and training in 8 motivational strategies to incorporate physical activity into daily routine
- Tips to overcome common barriers to physical activity
- Instruction on developing physical activity plan. Sample plans are provided for those considering walking, a combination of running and walking, stretching and strengthening, or keeping active through daily chores. All plans include downloadable forms.
- Education and training in how to properly warm up before aerobic activity and cool down afterward.
- Interactive logbook that calculates calories expended, charts progress, and records activities. Logbook is downloadable.
- Information about the motivational advantages of engaging in physical activity in groups, teams, or clubs
- Education and training in 7 strategies to prevent injury.
- Education about the role of diet and alcohol in an active lifestyle
- Tips for buying the right kinds of clothing and gear including
  - Basic equipment needs
  - Home gym equipment

Food Smart (Diet/Nutrition*)*

How Food Smart is Your Eating?

- Interactive Assessment of daily calorie and fat intake
- Calculation of Body Mass Index (BMI)
- Assessment of user's daily food categories based on the *Healthy Eating Pyramid*

Food Smart Facts and Guidelines

- Why be food smart?
- Video testimonials -- benefits of good nutrition and weight management
- Information on popular diets (*Atkins*, *South Beach*, etc.)
- Detailed explanation of Food Smart adaptation of the *Healthy Eating Pyramid* developed by Walter Willett, M.D. at Harvard Medical School
- Carbohydrates – overview of the role of carbohydrates in a healthful diet
- Fats – overview of the role of fats, types of fats, and tips for healthful choices
- Proteins – overview of proteins, food sources, and tips for healthful choices
- Hydration needs and tips for the selection of healthful beverages
- Alcohol guidelines for drinkers who can safely drink alcohol
- Interactive exercise in healthful meal selection
- Information and training in reading the Nutrition Facts Label
    - Demonstration of using the label to check out fat content
    - Trans-fats and the nutrition facts label
- Information and training in reading the Ingredients List
- Information and training in using the nutrition facts label and ingredients list to evaluate carbohydrates.
- Answers to frequently asked questions regarding nutrition, weight management, health, and disease prevention

Strategies for Success

- Guidelines for setting food SMART goals – Specific, Measurable, Achievable, Rewarding, and Timed goals – with examples of each.
- Setting goals based on personal assessment information
- Printable smart goal planner and progress trackers
- Interactive daily meal planning exercises
- Information and tips for satiation
- Shopping strategies and tips
- Staying on the path – interactive videos demonstrating the following behavioral strategies (with accompanying, printable tip sheets)
    - Planning ahead for healthy snacks
    - Refusing unwanted drinks or food
    - Using portion control with favorite, rich foods
    - Selecting the most healthful cooking method
- Tracking obstacles and strategies that work, with printable tracker
    - Behavioral tips for avoiding overeating

Stress Management

Assess Your Stress

- Self-assessment of the physical, emotional, and behavioral signs of stress including the presence of anxiety or depression
- Self-assessment of effective coping strategies

The Stress Response

- Information about of the physiological basis of stress including
  - The usefulness of stress to motivate activity
  - The evolutionary history of our stress system
  - The deleterious effects of chronic stress
- Description of the reciprocal nature of bad coping choices and increased stress levels

Identify Stressors

- Education and training in the 5 steps of effective stress management
- Information about the physical, emotional, and behavioral signs of stress
- Instruction on keeping a stress journal including a downloadable journal

Avoid Negative Coping

- Information on the ineffectiveness of temporary escapes from stress (e.g., overeating, smoking, alcohol)
- Special focus on the relationship between alcohol use and managing stress including:
  - Recommendations for moderate alcohol use
  - Self-assessment of alcohol use
  - The trap of alcohol dependence
  - Replacing alcohol with positive alternatives to manage stress
  - Tips for cutting down alcohol use including how to manage social drinking

Make Positive Choices

- Education and training in numerous proven strategies to reduce stress including
  - Interactive exercises designed to demonstrate the influence of negative thinking biases that increase stress followed by training to evaluate and change these interpretive errors.
  - Adding humor and laughter to daily routine
  - Time management techniques
  - Assertiveness training
  - The importance of social support to meet both instrumental and emotional needs
  - Effective problem-solving strategies
  - The importance of daily physical activity
  - Relaxation techniques such as progressive muscle relaxation, diaphragmatic breathing, guided imagery, and meditation.
    - Practice and mastery of each technique is facilitated by downloadable instruction sheets and audio MP3 files with guided instruction

Resources

- Extensive links to outside sources of information are provided
- Citations of work which form the basis of this program are included for further study
