# Supplementary material for: A Field Test of a Web-Based Workplace Health Promotion Program to Improve Dietary Practices, Reduce Stress, and Increase Physical Activity: Randomized Controlled Trial
Source: J Med Internet Res. 2007 Jun 19;9(2):e17. doi: 10.2196/jmir.9.2.e17 (PMC1913939; doi:10.2196/jmir.9.2.e17)
Supplement: Supplementary file 3 [file jmir_v9i2e17_app3.pdf]

Cook RF, Billings DW, Hersch RK, Back AS, Hendrickson A
A Field Test of a Web-Based Workplace Health Promotion Program to Improve Dietary Practices, Reduce Stress, and Increase Physical Activity: Randomized Controlled Trial
J Med Internet Res 2007;9(2):e17
<URL: http://www.jmir.org/2007/2/e17/>

Multimedia Appendix 3

Print Materials
Outline of Content

Fitness: The High Performance Lifestyle
StayWell/Krames © 1998

I. Fitness: Are You Ready?
   a. Why Should You be Active?
   b. Stages of Change Model
      i. Which stage are you at?
      ii. Pre-Contemplation stage
      iii. Contemplation stage
         1. Decrease your "cons" list
         2. Increase your "pros" list
   c. Cons-My Reasons for Not Being Active
   d. Pros-My Reasons for Being Active
      i. Would you like to enjoy:
      ii. Other personal benefits might include:
      iii. Preparation stage:
      iv. Action stage:
      v. Maintenance stage:
      vi. Termination stage
   e. Graphic with sections the reader can complete
      i. Ways to Find Time For Exercise
      ii. Ways to Make Exercise More Convenient
      iii. Ways to Be More Active at Home
      iv. Ways to Be More Active at Work

II. The F.I.T. Prescription: Skill 1
   a. The New Fitness Prescription
      i. The "traditional" F.I.T. prescription recommended:
         1. Frequency
         2. Intensity
         3. Time
      ii. The "new" F.I.T. prescription for cardiovascular fitness recommends:
         1. Frequency
         2. Intensity Time
   b. Should You See A Physician Before Beginning an Exercise Program?
      i. 7 questions for reader to answer to determine whether he/she should see a physician before starting an exercise program
      ii. Graphic of Target Heart Range Chart
   c. Exercising at the Right Pace
      i. Graphic of BORG Scale of Perceived Exertion
   d. Warming Up/Cooling Down
   e. Do You Know When to Stop?
      i. Graphic of Warm Up, Aerobic Activity, Cool Down

III. Aerobic Activity: Skill 2

a. How much aerobic activity is necessary to be healthy?
    i. Graphic: How Active Are Americans?
b. Do You Know How Fit You Are?
    i. A Simple Walking Test
    ii. Equipment Needed
    iii. Graphic of Exercise Tracking Chart: Date/Time/Heart Rate

IV. Flexibility: Skill 3

a. Proper Stretching Can:
b. The Rules of Stretching
    i. Graphic of a person performing a variety of stretches

V. Strength-Building Activities: Skill 4

a. The F.I.T. Prescription for Building Strength
    i. Intensity
    ii. Time
    iii. Graphic of person performing a variety of exercises

VI. The S.M.A.R.T. Plan: Skill 5

a. Specific
b. Measurable
c. Agreed Upon
d. Rewarding
e. Trackable
f. Hints to Help You Stay on Track
g. Graphic of a SMART plan Contract the reader can fill out

VII. References

Stress Management: Personal Action Guide
StayWell/Krames © 1999

I. Intro
  a. Everyone Has Stress
    i. The Pros and Cons of Stress
  b. What's Ahead (graphic telling each of the five skills to be covered in the booklet)
  c. Starting to Manage Stress
    i. Finding a Place to Start
    ii. Learning to Deal with Stress

II. Skill 1: Learning About Stress: How does stress affect your body, mind, and emotions?
  a. How Your Body Reacts to Stress
  b. How You May Respond to Stress
    i. Physical Symptoms (checklist of symptoms underneath)
    ii. Emotions (checklist of symptoms underneath)
    iii. Thinking (checklist of symptoms underneath)
    iv. Behavior (checklist of symptoms underneath)

III. Skill 2: Getting to Know Your Stressors: What triggers your stress? It's time to find out.
  a. What Can Trigger Your Stress Response?
    i. Common Stressors (examples underneath)
  b. Keeping Track of Your Stressors
    i. Graphic of Stress Log: Date/Time, Stressor, Response

IV. Skill 3: Taking Action: How You Respond to a Stressor is Your Choice
  a. Choosing Your Response to a Stressor
    i. Accepting a Stressor
      1. Activity-Reader can write down a stressor from previous page that he/she can't control and how he/she will accept it
    ii. Avoiding a Stressor
      1. Activity-Reader can write down a stressor from previous page that he/she can avoid & how he/she will do so
    iii. Altering a Stressor
      1. Graphic where reader can write down a stressor from previous page that he/she can alter & how he/she will do so
    iv. Adapting to a Stressor
      1. Activity-Reader can write down a stressor from previous page that he/she can adapt to & how he/she will do so
      2. How Will You Respond to Stress? Exercise for reader.

V. Skill 4: Preventing and Relieving Stress: To keep stress away, take some time to manage your day

a. Managing Your Time
b. Curbing Stress with Activity and Relaxation
    i. Aerobic Exercise
    ii. Stretching and Yoga
    iii. Stress Busting Activity Tips
    iv. Relaxation Techniques
        1. Deep breathing
        2. Progressive relaxation
        3. Stress-Busting Relaxation Tips

VI. Skill 5: Setting SMART Goals

a. Specific
b. Measurable
c. Achievable
d. Rewarding
e. Trackable
f. Break Though Your Barriers
    i. Activity-Common barriers to managing stress and tips to get past them (boxes of 4 barriers with tips and a spot where the reader can write in their own way).

VII. Staying on Track

Low Fat Eating
StayWell/Krames © 1993, 1997, 1998, 2001, 2002

I. What is Dietary Fat?
  a. Why Eat Less Fat?
  b. How to Get Started

II. Do You Eat Too Much Fat?
  a. Picture of a checklist about food habits & preferences & a picture of a check box with comments about the checklist "The more boxes you checked…"

III. Can Fat Hurt Your Health?
  a. Overweight
  b. Heart Disease
  c. High Blood Pressure
  d. Cancer
  e. Diabetes

IV. How Much Fat is Enough?
  a. Chart "Daily Grams of Fat"
  b. How to Read This Chart

V. The Food Pyramid
  a. Picture of the Food Pyramid with description of
    i. Fat, Oils, & Sweets Group
    ii. Milk, Yogurt, & Cheese Group
    iii. Vegetable Group
    iv. Meat, Poultry, Fish, Dry Beans, Eggs, & Nuts Group
    v. Fruit Group
    vi. Bread, Cereal, Rice, & Pasta Group
  b. Chart: "What Counts as a Serving?"
  c. Tips for Measuring

VI. Where's the Fat?
  a. Choosing Low-Fat Foods
  b. Avoiding High Fat Foods
  c. Types of Fat
    i. Monounsaturated fats
    ii. Polyunsaturated fats
    iii. Saturated fats
    iv. Hydrogenated oils

VII. Reading Labels Helps Find Fat
  a. Picture of a Nutrition Chart with description of what different sections mean
    i. Serving Size
    ii. Calorie Information
    iii. Total Fat
    iv. Types of Fat
    v. Ingredients
  b. Track Your Fat

VIII. Low-Fat Shopping
  a. Starches and Grains
  b. Vegetables and Fruits
  c. Dairy
  d. Meat and Fish

IX. Low-Fat Cooking
  a. Preparation
  b. Changing Recipes

X. Low-Fat Eating Out
  a. Boxes describing with recommendations when eating:
    i. Fast Food
    ii. Italian
    iii. Mexican
    iv. Asian

XI. Changing Your Ways
  a. Know Your Daily Fat Range
  b. Choose How You'll Change
  c. Take Action
  d. Reward Yourself When You Succeed

XII. Small Changes Add Up!
  a. Table of low-fat food substitutes & grams of fat in each
    i. Breakfast Foods
    ii. Lunch Foods
    iii. Dinner Foods
    iv. Snacks & Sweets

XIII. Enjoying a Healthy Lifestyle
  a. Resources

Getting Started with Weight Management: Learn the Facts, Avoid the Fads
StayWell/Krames © 1999

I. Why Lose Weight
  a. Reasons to Lose Weight
    i. For Yourself
    ii. For Your Health
  b. Fads Don't Work
  c. Getting the Most from This Booklet
    i. Describe the little bubbles throughout the packet:, Try this Tip, Avoid this Trap, Know this Fact, Ask your Doctor

II. What's Stopping You?
  a. Looking at Your Barriers
  b. Breaking Down Your Barriers

III. Set Your Long-term Goal
  a. Definition of a Healthy Body
  b. Choosing a Long-term Goal
  c. Another way to Measure Healthy Weight
    i. BMI chart

IV. Make an Action Plan
  a. Take Small Steps to Success
  b. Track Your Progress
  c. Reward Yourself

V. Healthy Eating
  a. What, When, Why, and How You Eat
  b. Eat Less Fat
  c. Understanding Food Labels
  d. Eat More Fiber
  e. Drink Plenty of Water

VI. Exercise and Activity
  a. The Benefits of Exercise
  b. Make Activity Part of Your Day
  c. Make Exercise Fun

VII. Stick With It
  a. Set Yourself Up for Success
  b. Believe That You Can Do It
  c. A Caution About Weight-Loss Programs

VIII. Getting Support
  a. Numbers for various health organizations

The Family Connection: Points for Parents
ISA Associates Inc. © 1996

I. The Family that Plays Together…
   a. Keep it light
   b. Be a beginner
   c. Adopt an experimental attitude
   d. Be patient as you try a new activity
   e. You don't have to be among the "super fit"
   f. Recognize and appreciate differences
   g. Active recreation can help blow off family stress…
      i. Creates a natural opportunity to show affection…
      ii. Family memories are made of this…
      iii. Remember-You set the example…
   h. Place for parents to complete the following
      i. Active fun we can try out as a family
      ii. Other ways we can relax together
      iii. Time we could set aside to just do nothing
      iv. Trade offs we might need to make (no fill in the blank for this one)

II. Keeping Your Kids Off Drugs-Specific Steps You Can Take
   a. Set a good example
   b. Give children a clear set of rules
   c. Be clear about rules regarding parties and driving
      i. If you drink don't drive; and
      ii. Never get into a car with a driver who is even remotely intoxicated.

Alcohol, Drugs, and a Healthy Lifestyle: What's the Connection?
ISA Associates Inc. © 1996

I. Intro

II. Healthy Alternatives to Feeling Good
   a. Relaxation and release from tension
   b. Release from inhibitions
   c. Feeling euphoria "high"
   d. Feeling stimulated, energized
   e. Altered states

III. The Drug Trap
   a. Intro
   b. Stimulant Drugs, Cocaine ("coke," "snow," "blow,")
   c. Methamphetamine ("crank," "speed," "monster," "Meth," "Ice")
   d. Opiates/Narcotics
   e. MDMA ("Ecstasy," "Adam")
   f. Marijuana/cannabis ("pot," "dope," "weed," "reefer")
   g. Other prescription drugs (this wasn't a main header like above but was bolded)

IV. Alcohol-A Legal Drug
   a. Heavy alcohol consumption is associated with a number of serious problems…
   b. Alcoholism (alcohol dependence) is a serious disease condition…
   c. Box telling recommended daily drinking amounts for men & women
   d. What is a "Drink"
   e. Checklist of the "Ripple Effect" (whether alcohol/drugs is getting in the way of healthy lifestyle)
   f. CAGE drinking screener with scoring information and recommendations of what to do for different scores
