# Supplementary material for: A Field Test of a Web-Based Workplace Health Promotion Program to Improve Dietary Practices, Reduce Stress, and Increase Physical Activity: Randomized Controlled Trial
Source: J Med Internet Res. 2007 Jun 19;9(2):e17. doi: 10.2196/jmir.9.2.e17 (PMC1913939; doi:10.2196/jmir.9.2.e17)
Supplement: Supplementary file 4 [file jmir_v9i2e17_app4.ppt]

## Slide 1
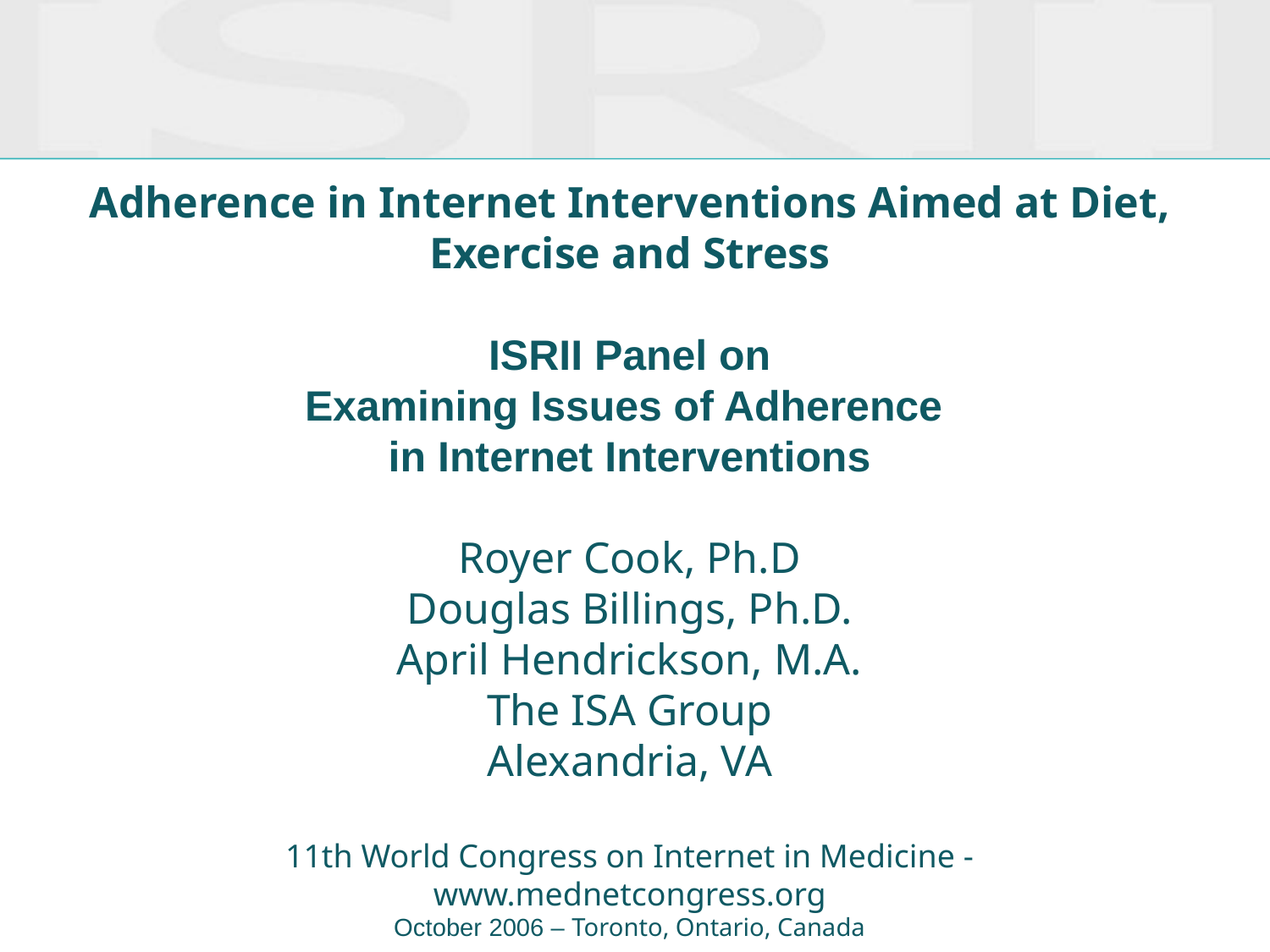

Adherence in Internet Interventions Aimed at Diet, Exercise and Stress
ISRII Panel on
Examining Issues of Adherence
in Internet Interventions
Royer Cook, Ph.D
Douglas Billings, Ph.D.
April Hendrickson, M.A.
The ISA Group
Alexandria, VA
11th World Congress on Internet in Medicine - www.mednetcongress.org
October 2006 – Toronto, Ontario, Canada

## Slide 2
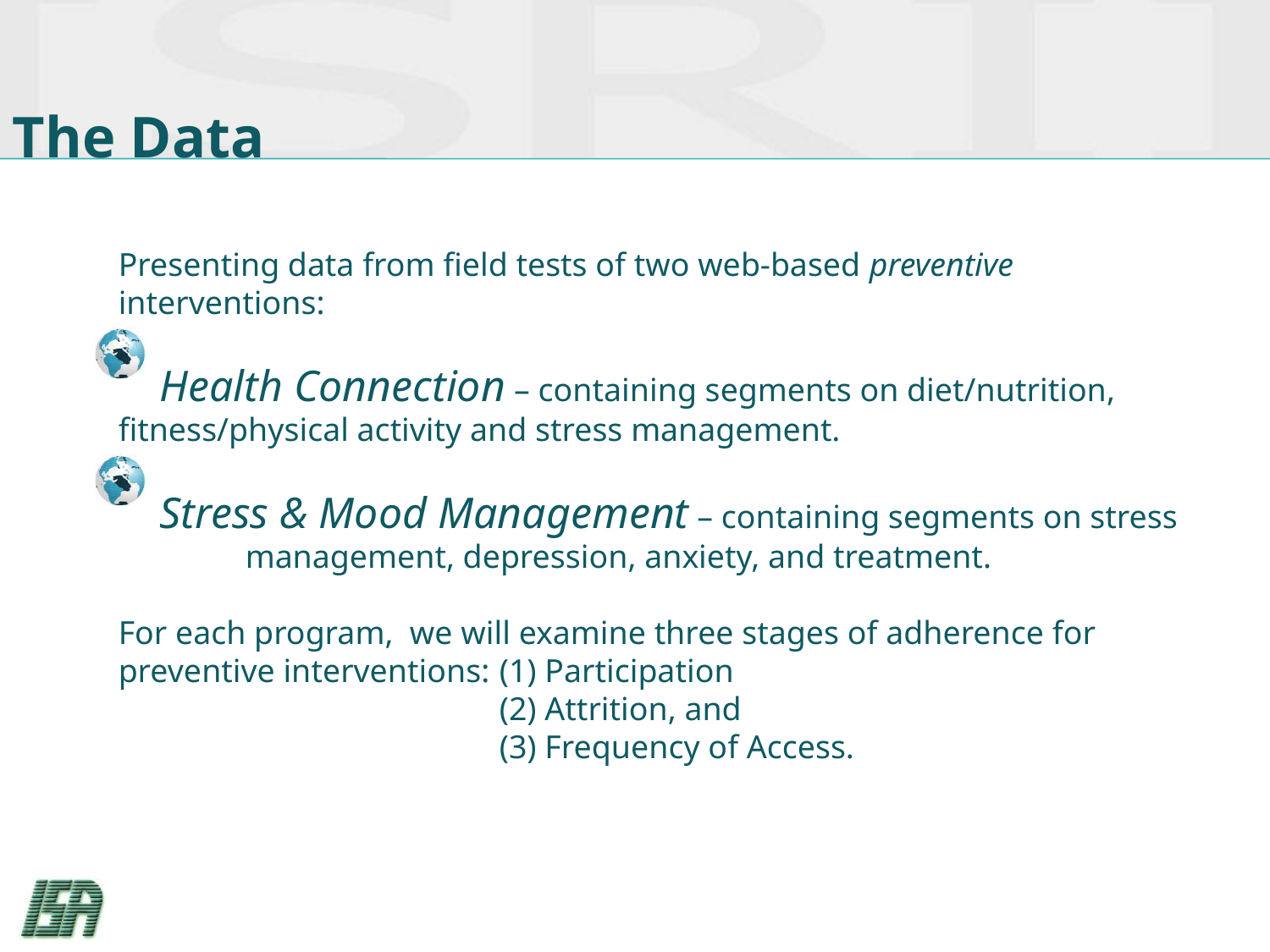

The Data
Presenting data from field tests of two web-based preventive interventions:
 Health Connection – containing segments on diet/nutrition, 	fitness/physical activity and stress management.
 Stress & Mood Management – containing segments on stress
	management, depression, anxiety, and treatment.
For each program, we will examine three stages of adherence for preventive interventions:	(1) Participation
			(2) Attrition, and
			(3) Frequency of Access.

## Slide 3
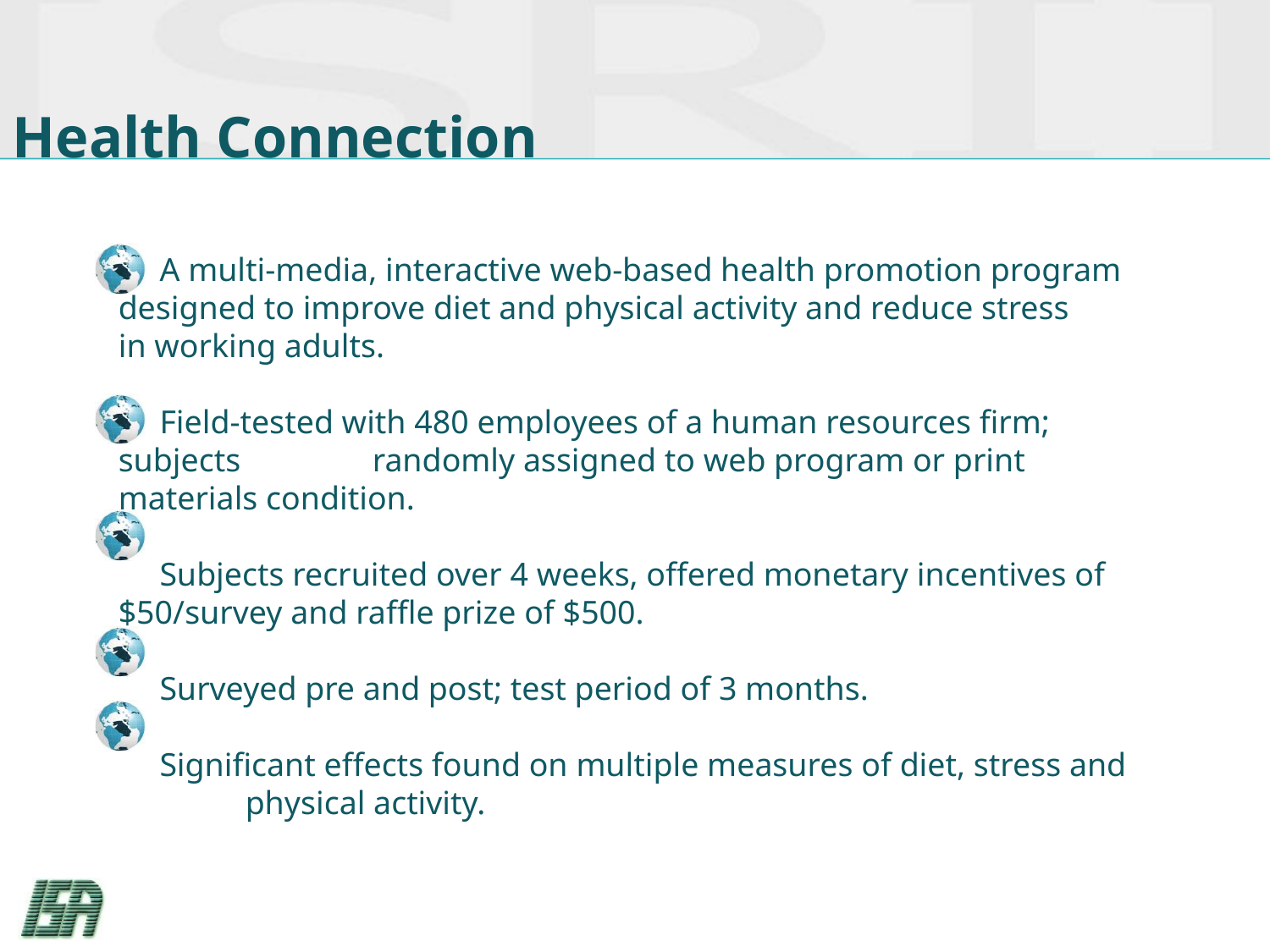

Health Connection
 A multi-media, interactive web-based health promotion program 	designed to improve diet and physical activity and reduce stress 	in working adults.
 Field-tested with 480 employees of a human resources firm; subjects 	randomly assigned to web program or print materials condition.
 Subjects recruited over 4 weeks, offered monetary incentives of 	$50/survey and raffle prize of $500.
 Surveyed pre and post; test period of 3 months.
 Significant effects found on multiple measures of diet, stress and 	physical activity.

## Slide 4
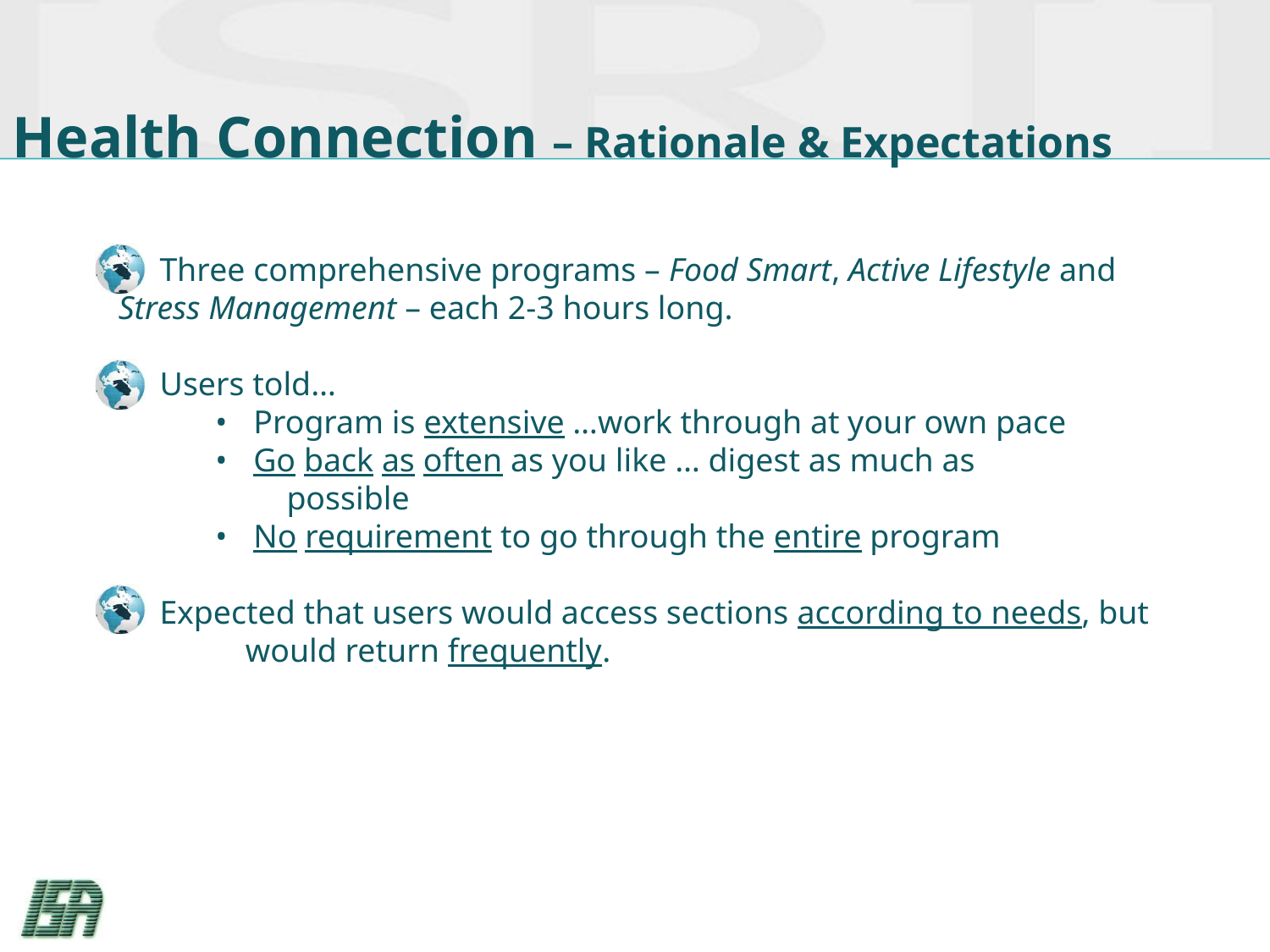

Health Connection – Rationale & Expectations
 Three comprehensive programs – Food Smart, Active Lifestyle and 	Stress Management – each 2-3 hours long.
 Users told…
 Program is extensive …work through at your own pace
 Go back as often as you like … digest as much as
	 possible
 No requirement to go through the entire program
 Expected that users would access sections according to needs, but 	would return frequently.

## Slide 5
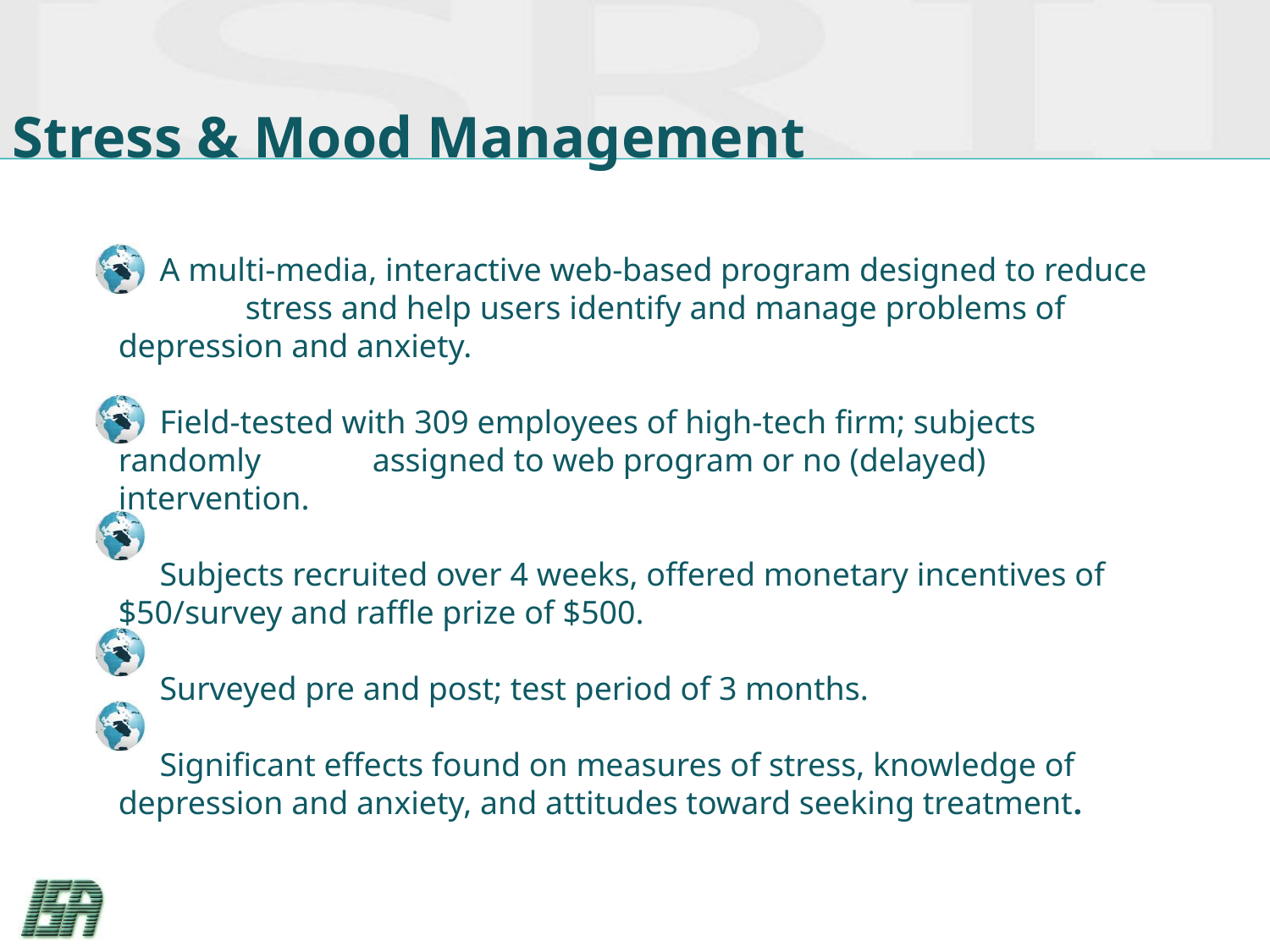

Stress & Mood Management
 A multi-media, interactive web-based program designed to reduce 	stress and help users identify and manage problems of 	depression and anxiety.
 Field-tested with 309 employees of high-tech firm; subjects randomly 	assigned to web program or no (delayed) intervention.
 Subjects recruited over 4 weeks, offered monetary incentives of 	$50/survey and raffle prize of $500.
 Surveyed pre and post; test period of 3 months.
 Significant effects found on measures of stress, knowledge of 	depression and anxiety, and attitudes toward seeking treatment.

## Slide 6
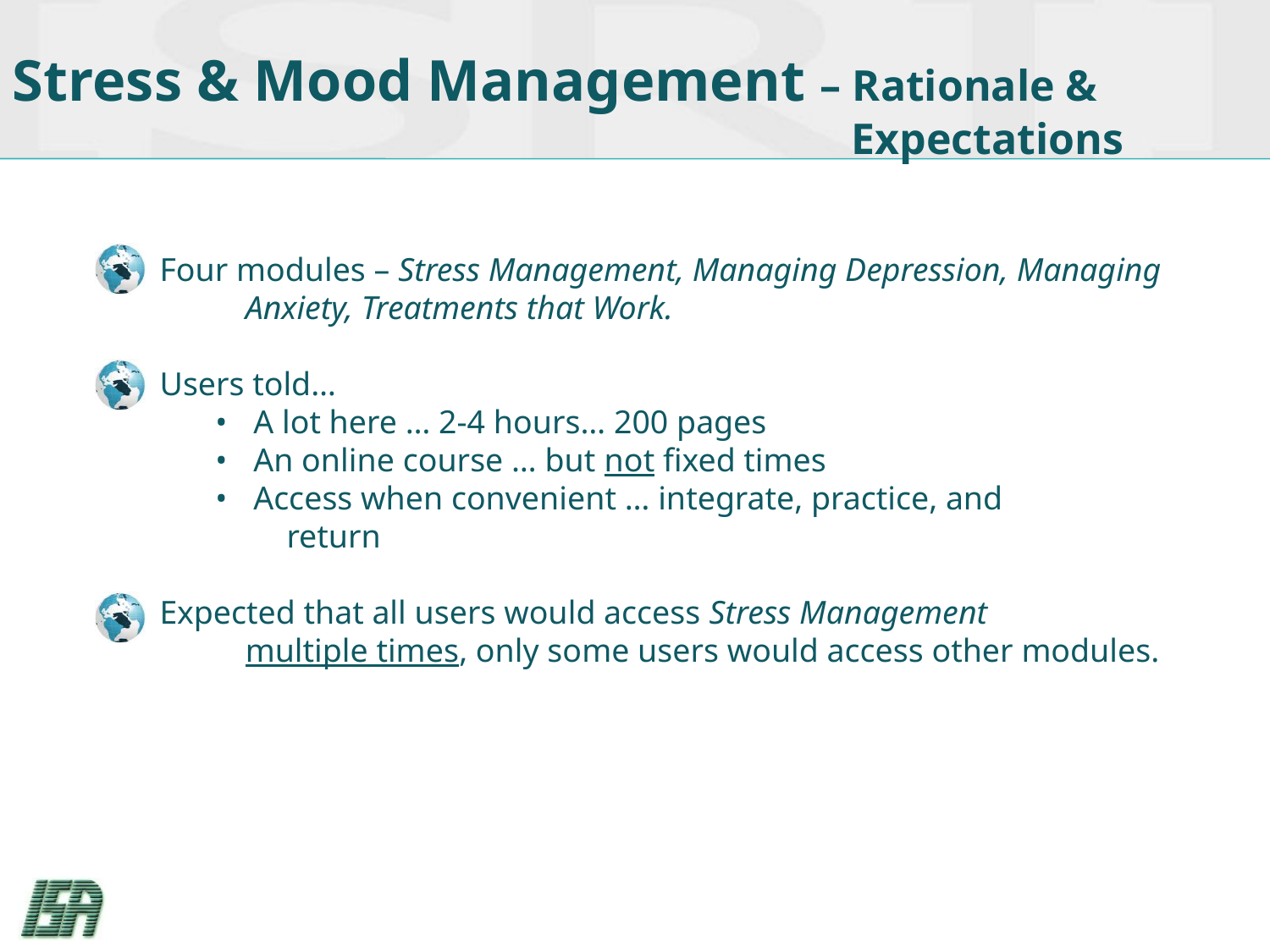

Stress & Mood Management – Rationale &
						 Expectations
 Four modules – Stress Management, Managing Depression, Managing 	Anxiety, Treatments that Work.
 Users told…
 A lot here … 2-4 hours… 200 pages
 An online course … but not fixed times
 Access when convenient … integrate, practice, and
	 return
 Expected that all users would access Stress Management
 	multiple times, only some users would access other modules.

## Slide 7
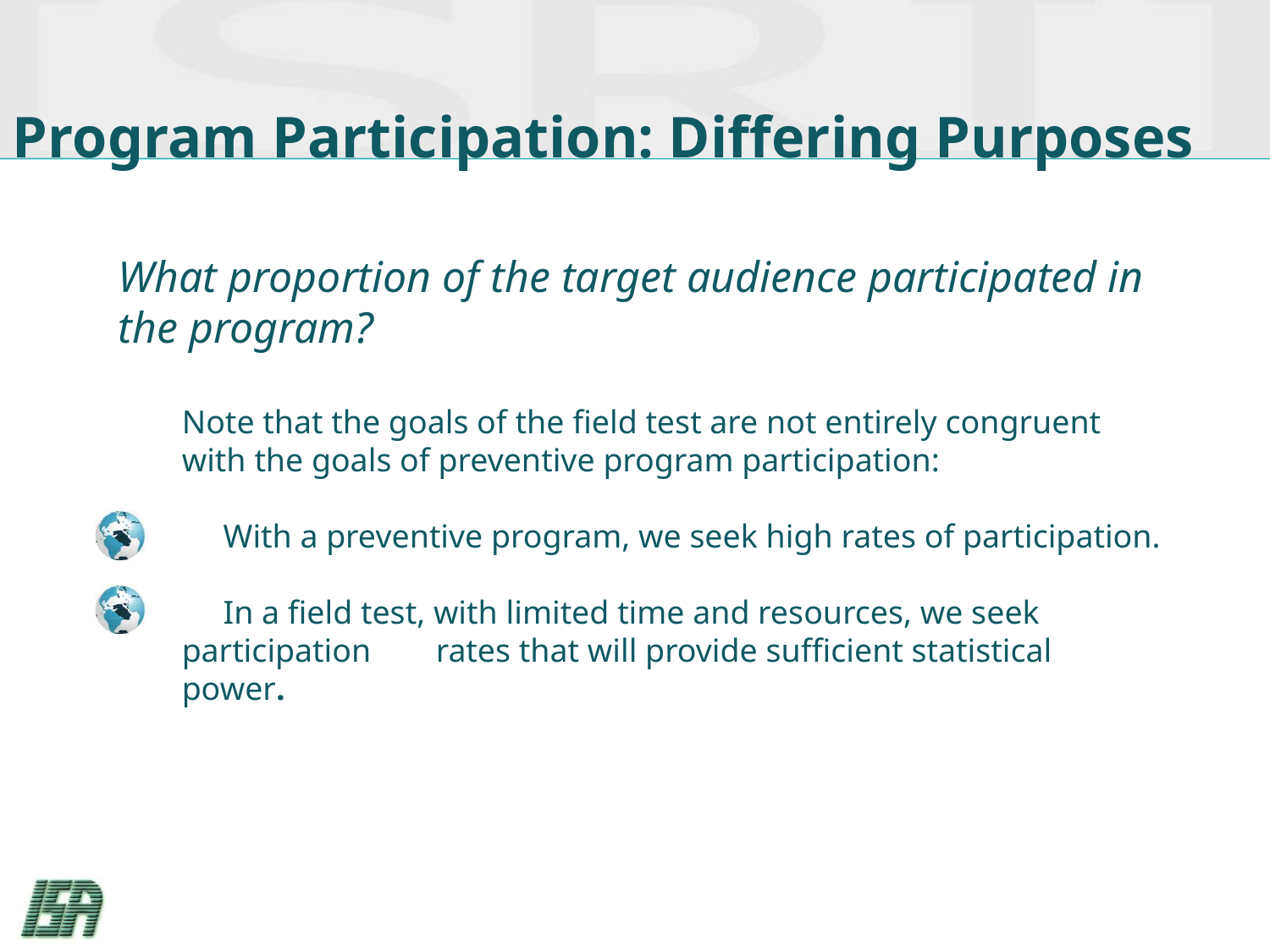

Program Participation: Differing Purposes
What proportion of the target audience participated in the program?
Note that the goals of the field test are not entirely congruent with the goals of preventive program participation:
 With a preventive program, we seek high rates of participation.
 In a field test, with limited time and resources, we seek participation 	rates that will provide sufficient statistical power.

## Slide 8
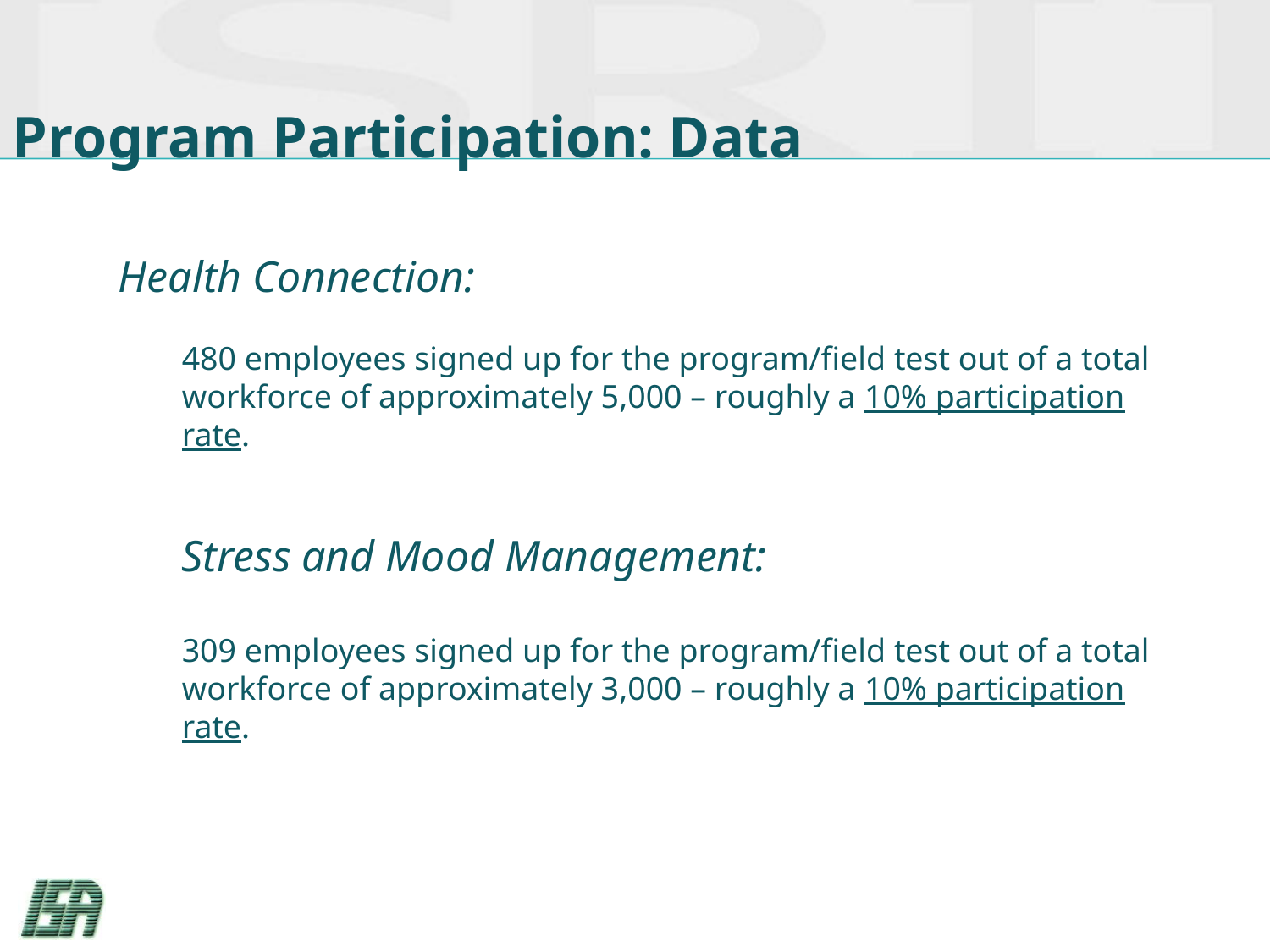

Program Participation: Data
Health Connection:
480 employees signed up for the program/field test out of a total workforce of approximately 5,000 – roughly a 10% participation rate.
Stress and Mood Management:
309 employees signed up for the program/field test out of a total workforce of approximately 3,000 – roughly a 10% participation rate.

## Slide 9
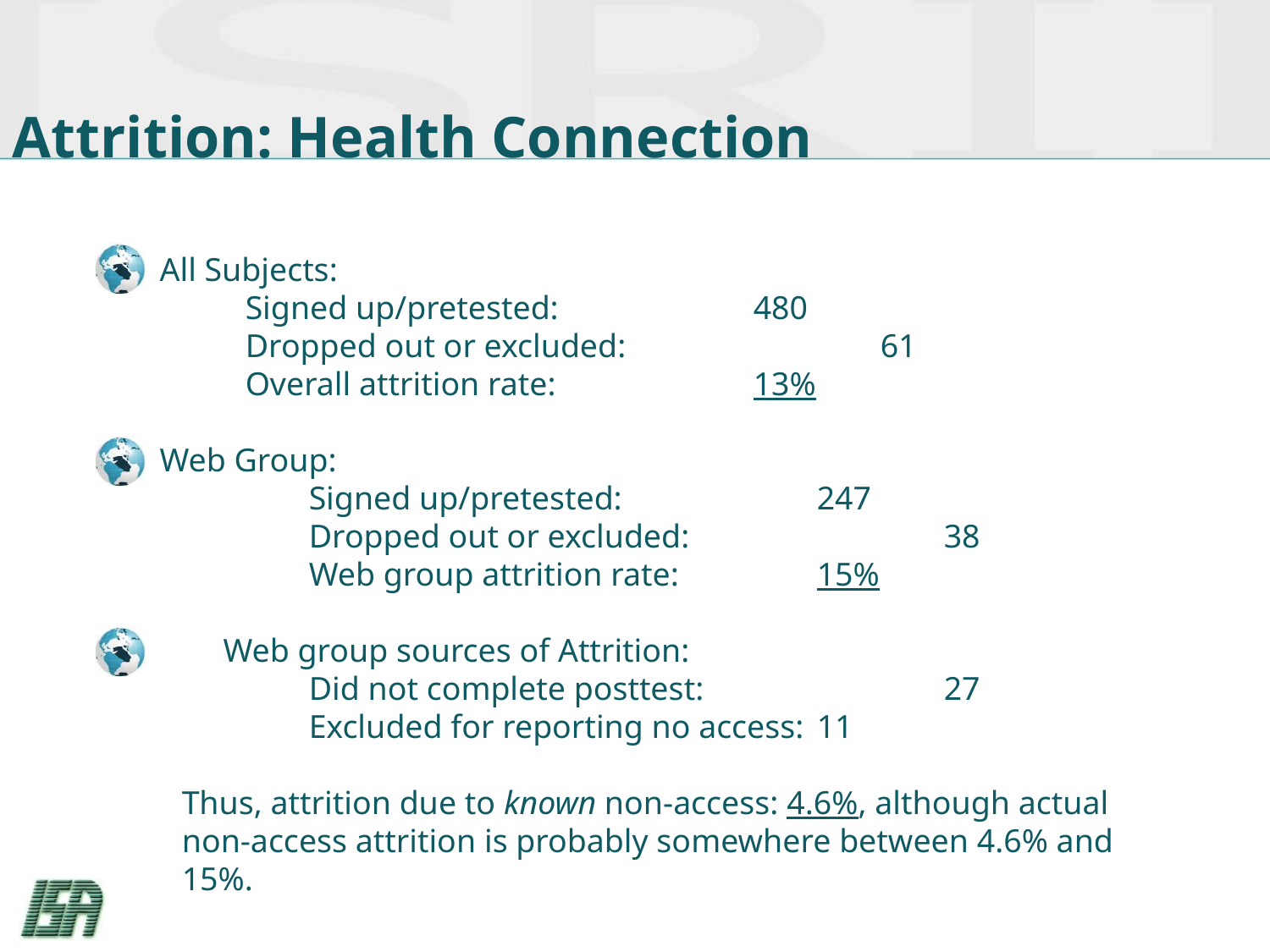

Attrition: Health Connection
 All Subjects:
	Signed up/pretested:		480
	Dropped out or excluded:		61
	Overall attrition rate:		13%
 Web Group:
	Signed up/pretested:		247
	Dropped out or excluded:		38
	Web group attrition rate:		15%
 Web group sources of Attrition:
 	Did not complete posttest:		27
 	Excluded for reporting no access:	11
Thus, attrition due to known non-access: 4.6%, although actual non-access attrition is probably somewhere between 4.6% and 15%.

## Slide 10
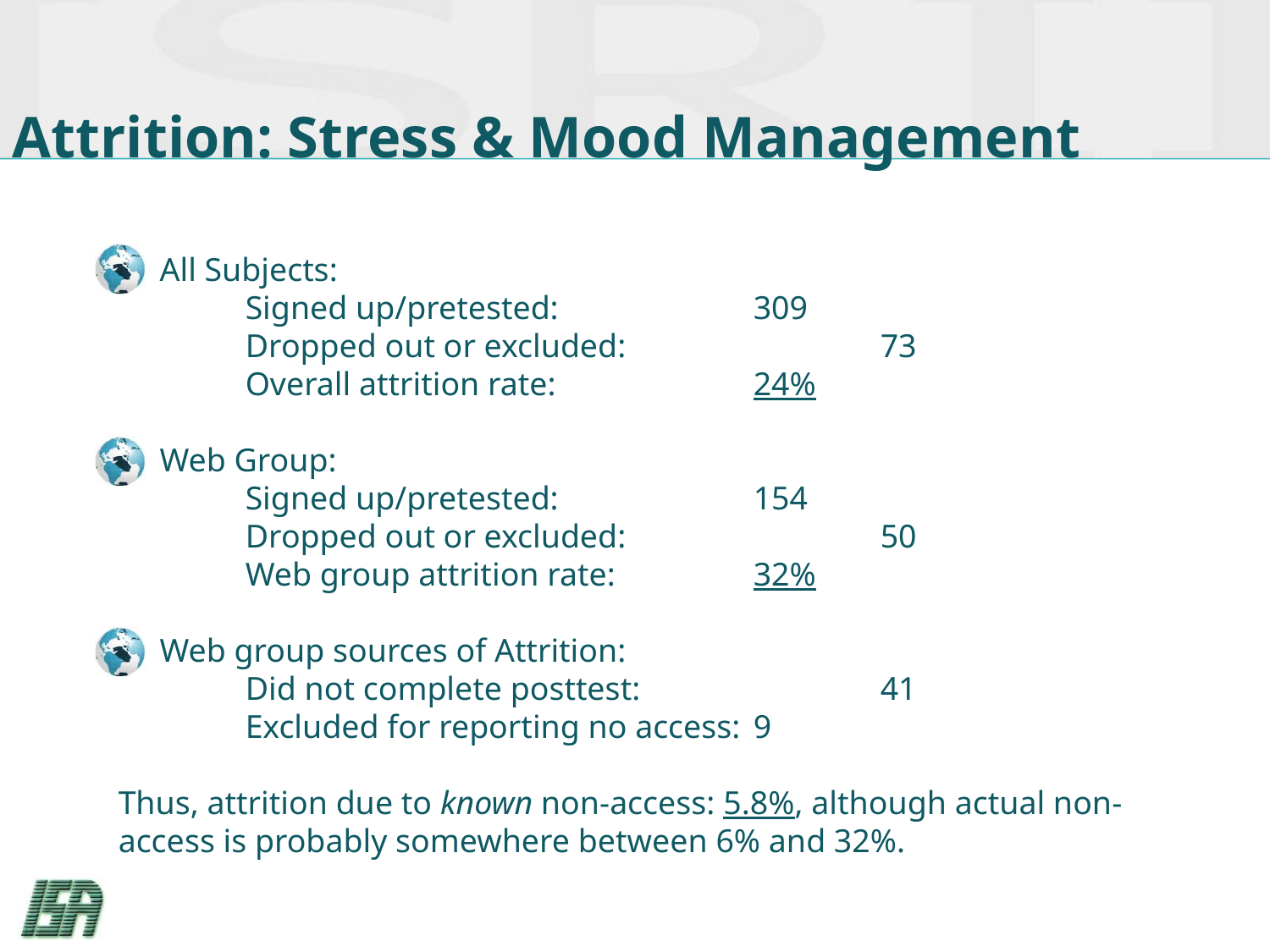

Attrition: Stress & Mood Management
 All Subjects:
	Signed up/pretested:		309
	Dropped out or excluded:		73
	Overall attrition rate:		24%
 Web Group:
	Signed up/pretested:		154
	Dropped out or excluded:		50
	Web group attrition rate:		32%
 Web group sources of Attrition:
	Did not complete posttest:		41
	Excluded for reporting no access:	9
Thus, attrition due to known non-access: 5.8%, although actual non-access is probably somewhere between 6% and 32%.

## Slide 11
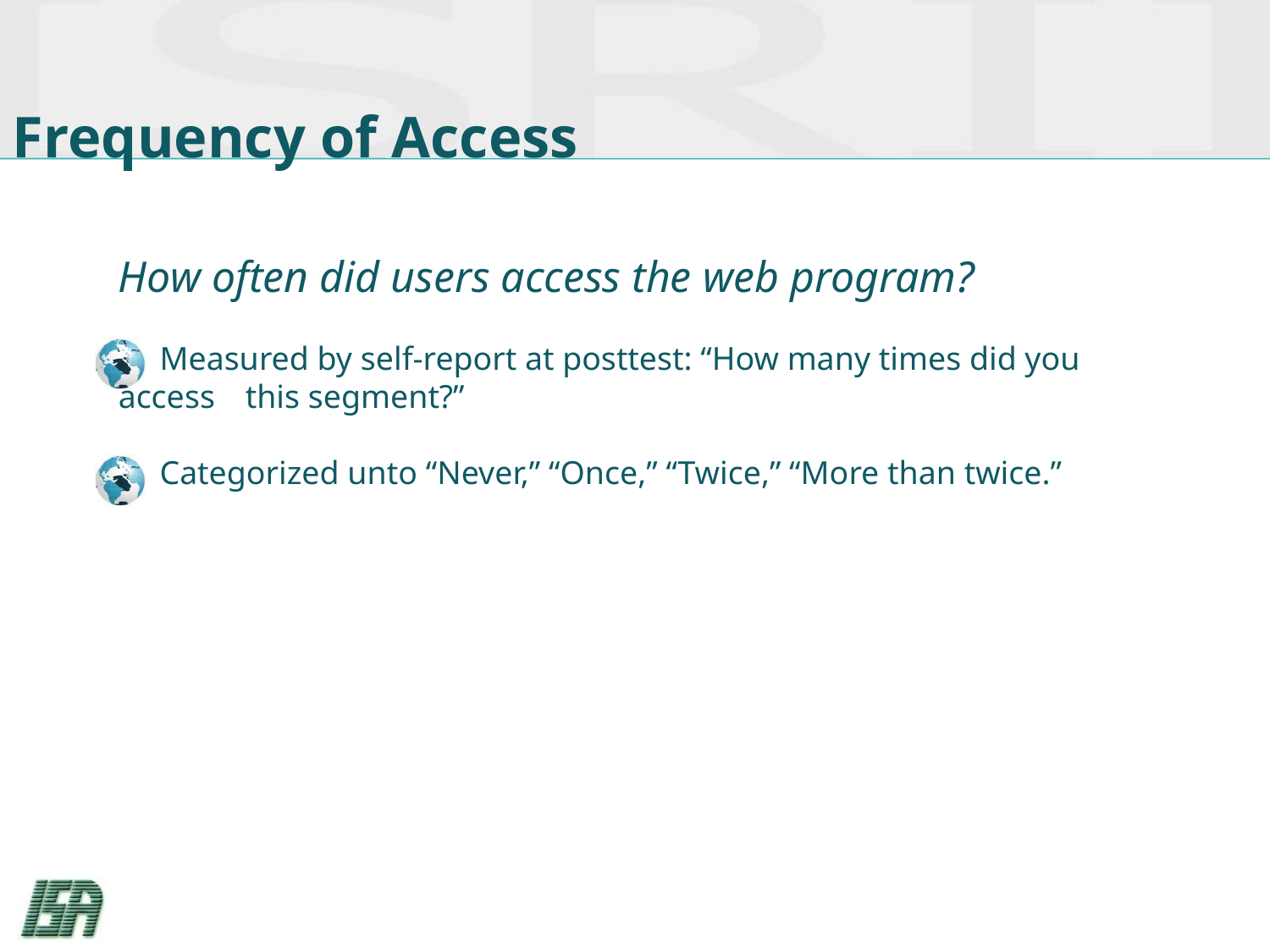

Frequency of Access
How often did users access the web program?
 Measured by self-report at posttest: “How many times did you access 	this segment?”
 Categorized unto “Never,” “Once,” “Twice,” “More than twice.”

## Slide 12
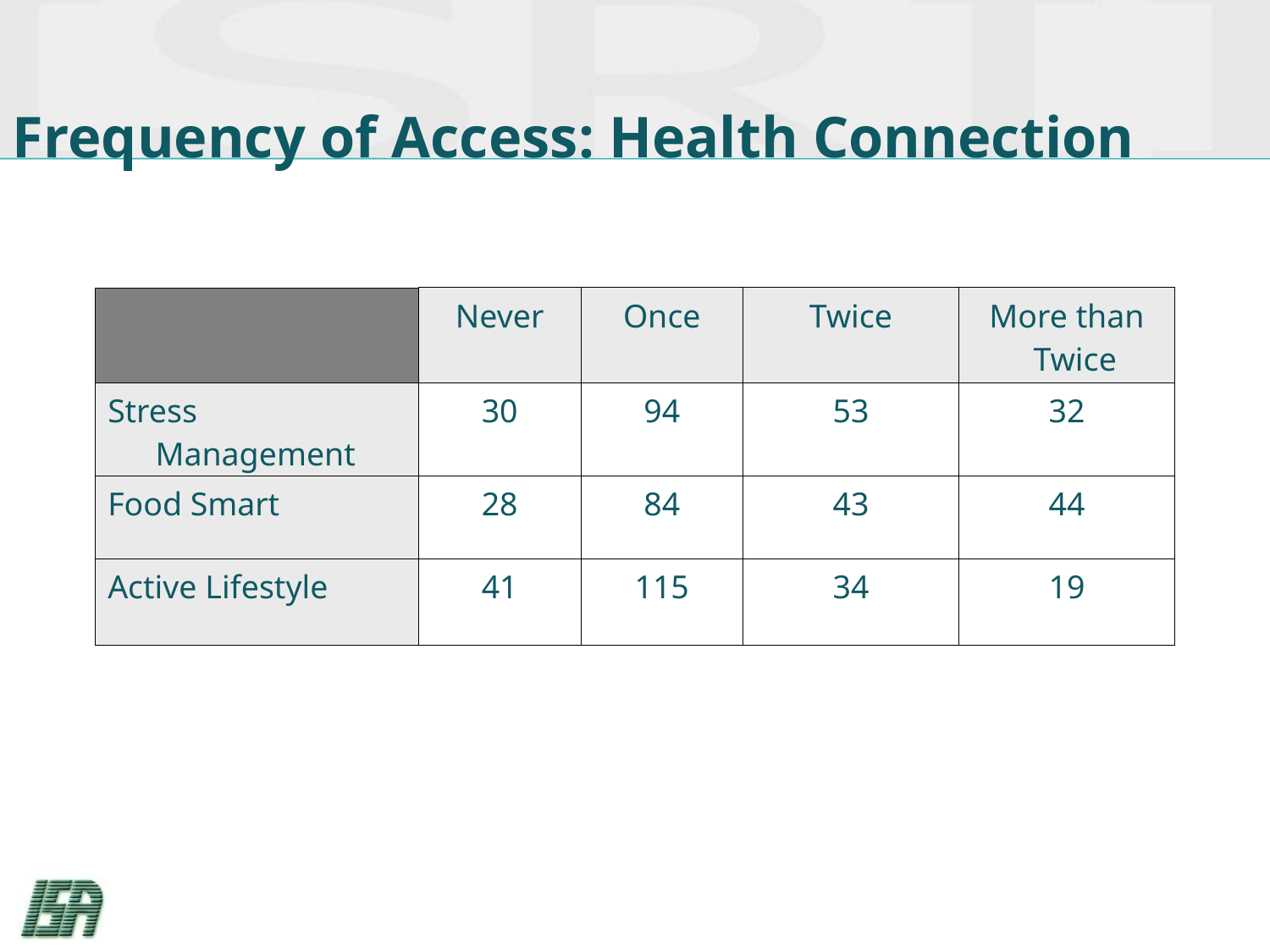

Frequency of Access: Health Connection
| | Never | Once | Twice | More than Twice |
| --- | --- | --- | --- | --- |
| Stress Management | 30 | 94 | 53 | 32 |
| Food Smart | 28 | 84 | 43 | 44 |
| Active Lifestyle | 41 | 115 | 34 | 19 |

## Slide 13
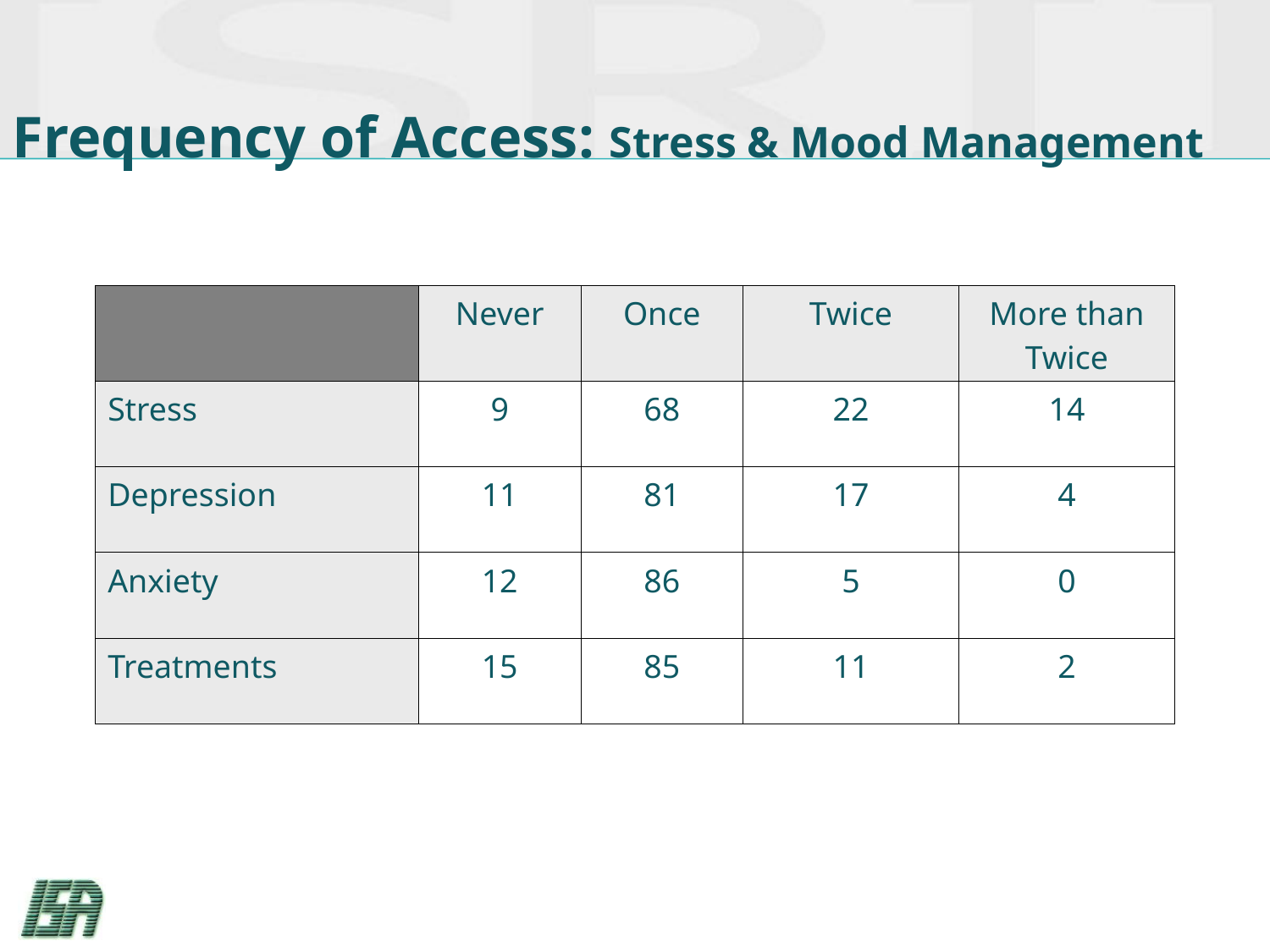

Frequency of Access: Stress & Mood Management
| | Never | Once | Twice | More than Twice |
| --- | --- | --- | --- | --- |
| Stress | 9 | 68 | 22 | 14 |
| Depression | 11 | 81 | 17 | 4 |
| Anxiety | 12 | 86 | 5 | 0 |
| Treatments | 15 | 85 | 11 | 2 |

## Slide 14
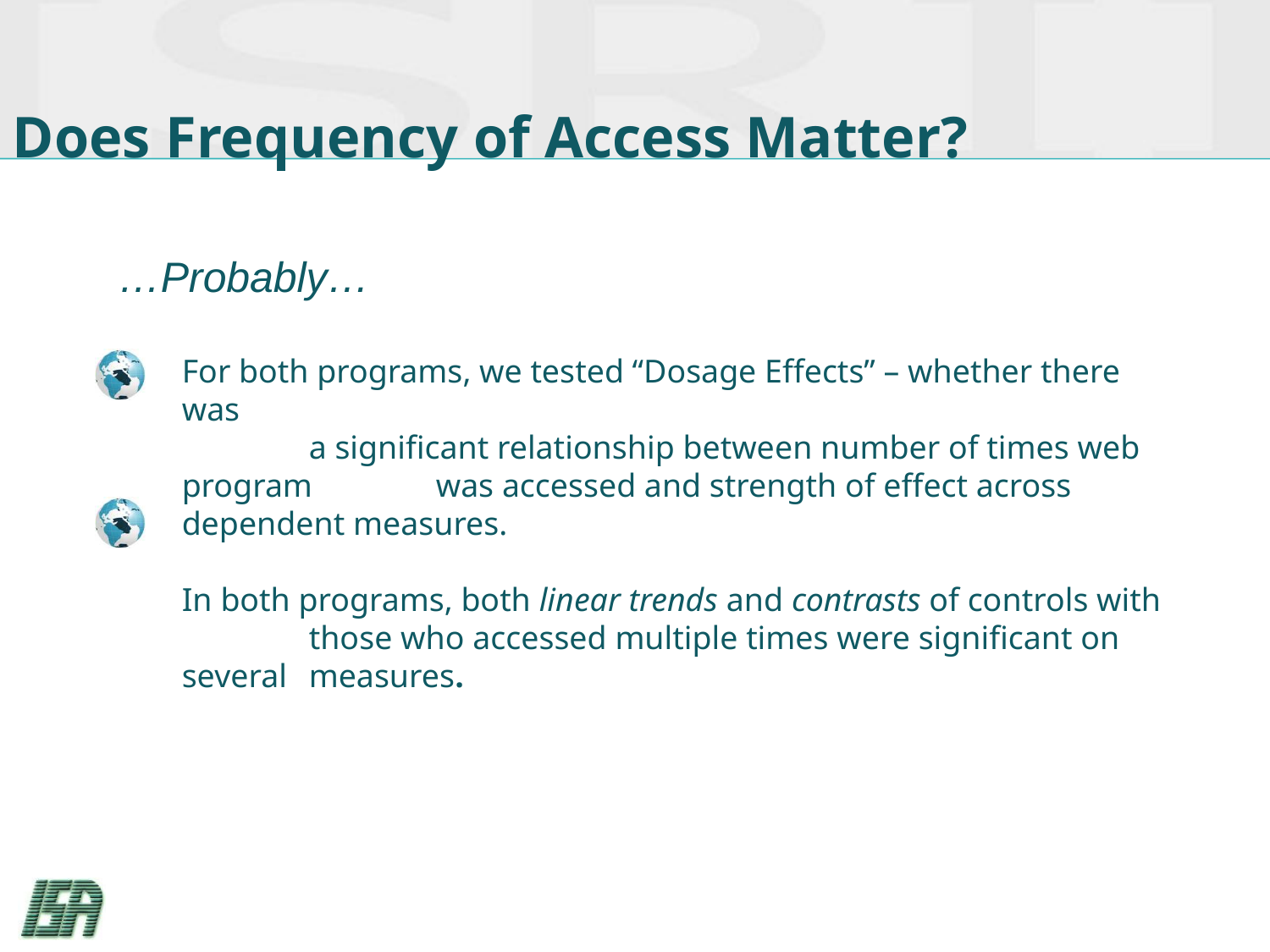

Does Frequency of Access Matter?
…Probably…
For both programs, we tested “Dosage Effects” – whether there was
	a significant relationship between number of times web program 	was accessed and strength of effect across dependent measures.
In both programs, both linear trends and contrasts of controls with 	those who accessed multiple times were significant on several 	measures.

## Slide 15
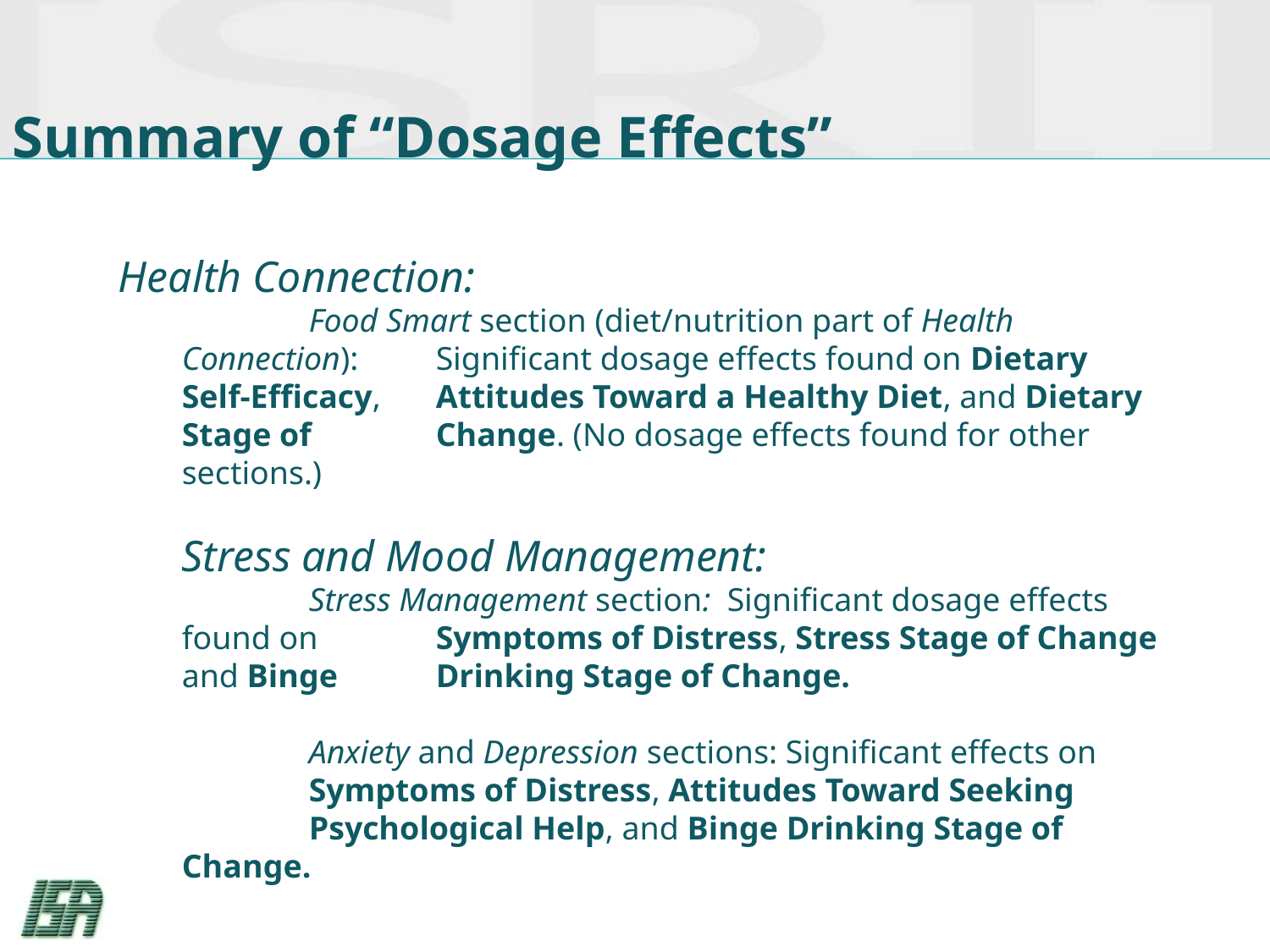

Summary of “Dosage Effects”
Health Connection:
	Food Smart section (diet/nutrition part of Health Connection): 	Significant dosage effects found on Dietary Self-Efficacy, 	Attitudes Toward a Healthy Diet, and Dietary Stage of 	Change. (No dosage effects found for other sections.)
Stress and Mood Management:
	Stress Management section: Significant dosage effects found on 	Symptoms of Distress, Stress Stage of Change and Binge 	Drinking Stage of Change.
	Anxiety and Depression sections: Significant effects on 	Symptoms of Distress, Attitudes Toward Seeking 	Psychological Help, and Binge Drinking Stage of Change.

## Slide 16
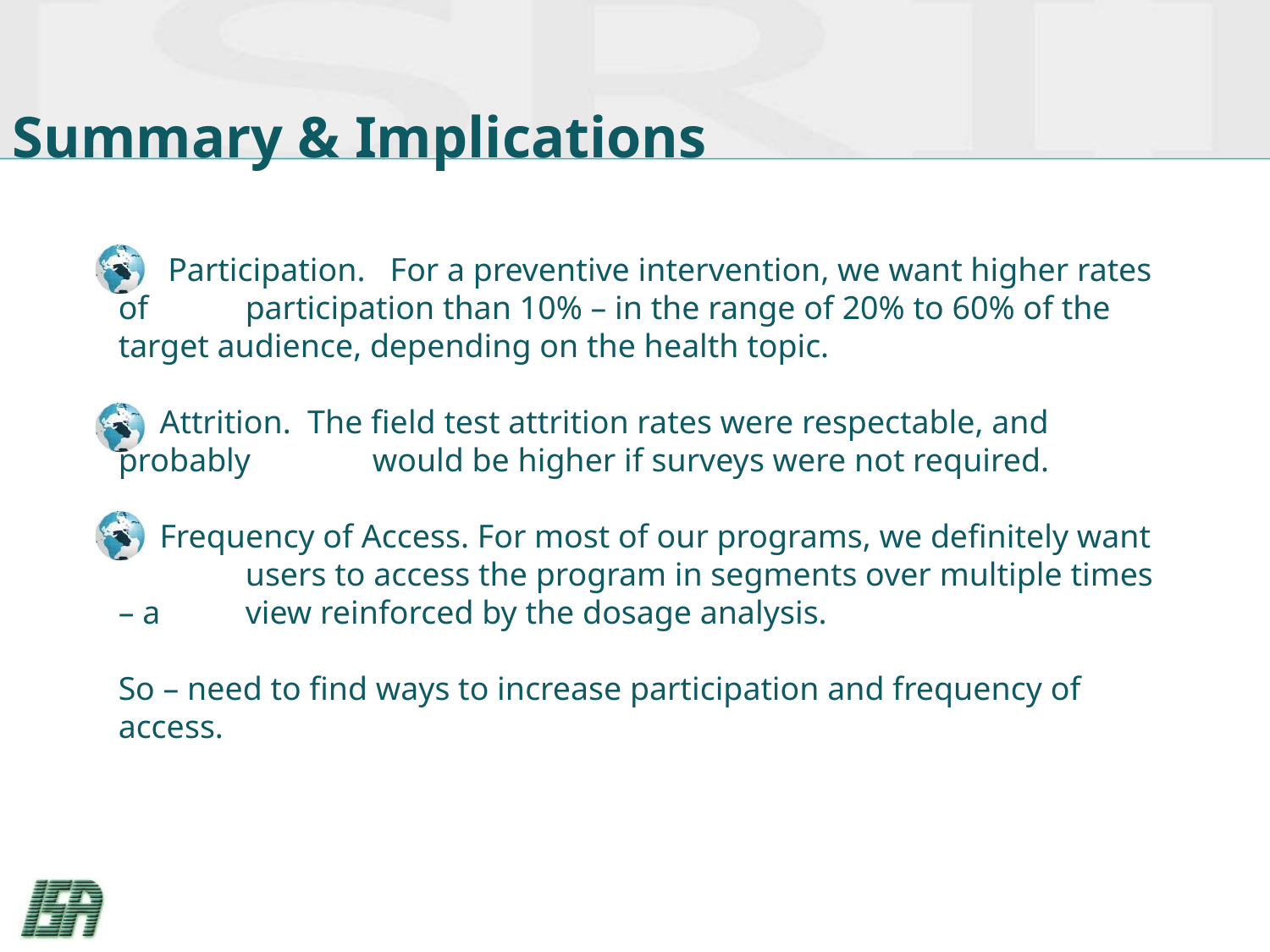

Summary & Implications
 Participation. For a preventive intervention, we want higher rates of 	participation than 10% – in the range of 20% to 60% of the 	target audience, depending on the health topic.
 Attrition. The field test attrition rates were respectable, and probably 	would be higher if surveys were not required.
 Frequency of Access. For most of our programs, we definitely want 	users to access the program in segments over multiple times – a 	view reinforced by the dosage analysis.
So – need to find ways to increase participation and frequency of access.

## Slide 17
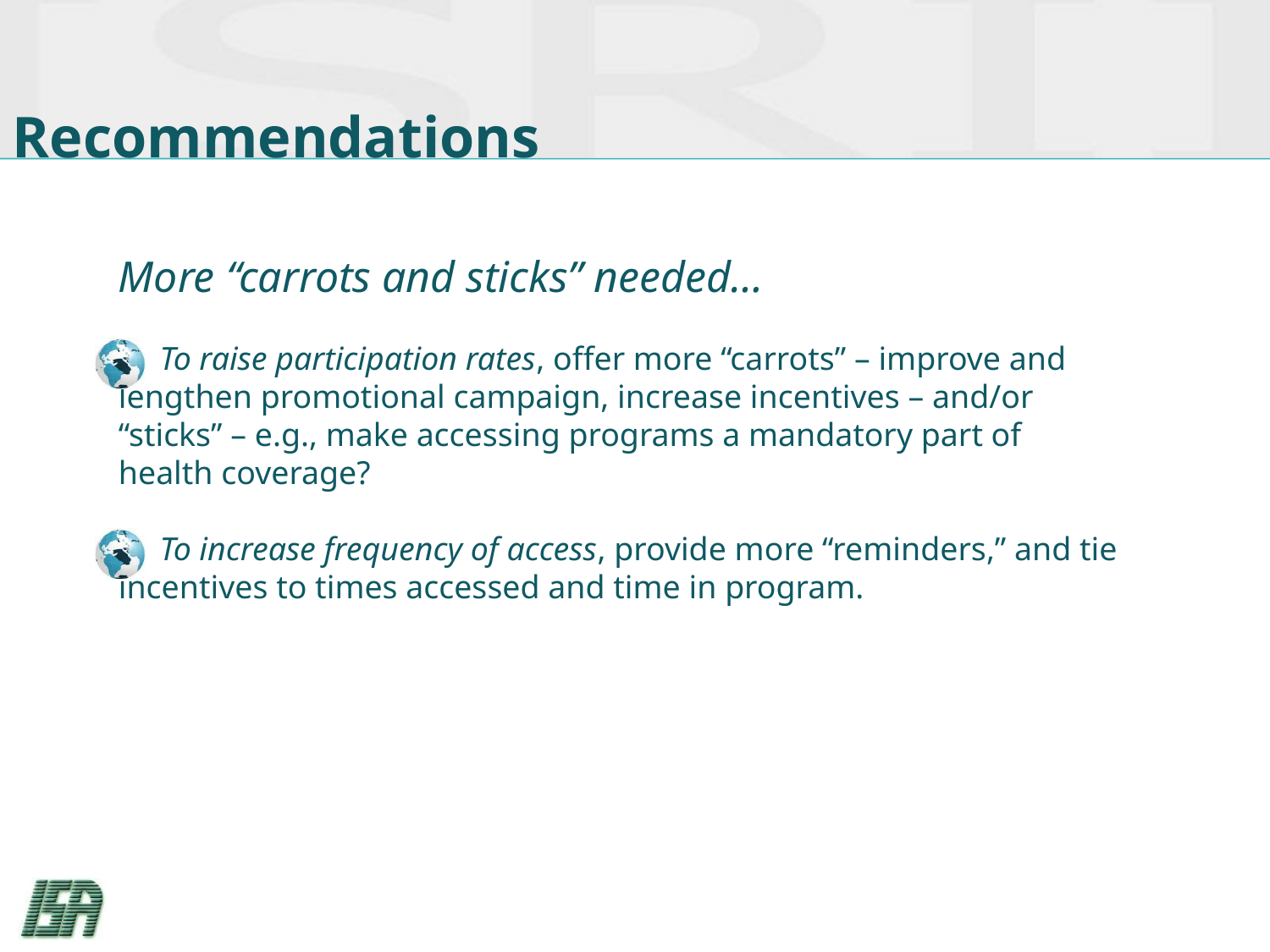

Recommendations
More “carrots and sticks” needed…
 To raise participation rates, offer more “carrots” – improve and 	lengthen promotional campaign, increase incentives – and/or 	“sticks” – e.g., make accessing programs a mandatory part of 	health coverage?
 To increase frequency of access, provide more “reminders,” and tie 	incentives to times accessed and time in program.
